# Metabolic signatures of birthweight in 18 288 adolescents and adults

**DOI:** 10.1093/ije/dyw255

**Published:** 2016-11-07

**Authors:** Peter Würtz, Qin Wang, Marjo Niironen, Tuulia Tynkkynen, Mika Tiainen, Fotios Drenos, Antti J Kangas, Pasi Soininen, Michael R Skilton, Kauko Heikkilä, Anneli Pouta, Mika Kähönen, Terho Lehtimäki, Richard J Rose, Eero Kajantie, Markus Perola, Jaakko Kaprio, Johan G Eriksson, Olli T Raitakari, Debbie A Lawlor, George Davey Smith, Marjo-Riitta Järvelin, Mika Ala-Korpela, Kirsi Auro

**Affiliations:** 1 Computational Medicine, Faculty of Medicine, University of Oulu and Biocenter Oulu, Oulu, Finland; 2 NMR Metabolomics Laboratory School of Pharmacy, University of Eastern Finland, Kuopio, Finland; 3 Department of Genomics and Biomarkers, National Institute for Health and Welfare, Helsinki, Finland; 4 Institute for Molecular Medicine Finland, University of Helsinki, Helsinki, Finland; 5 Medical Research Council Integrative Epidemiology Unit, University of Bristol, Bristol, UK; 6 School of Social and Community Medicine, University of Bristol, Bristol, UK; 7 Boden Institute of Obesity, Nutrition, Exercise, and Eating Disorders, University of Sydney, Sydney, NSW, Australia; 8 Department of Public Health, University of Helsinki, Helsinki, Finland; 9 Center for Life Course Health Research and Biocenter Oulu, University of Oulu, Oulu, Finland; 10 Department of Children, Young People and Families, National Institute for Health and Welfare, Oulu, Finland; 11 Department of Clinical Physiology, University of Tampere and Tampere University Hospital, Tampere, Finland; 12 Department of Clinical Chemistry, Fimlab Laboratories and School of Medicine, University of Tampere, Tampere, Finland; 13 Department of Psychological and Brain Sciences, Indiana University, Bloomington, IN, USA; 14 Children’s Hospital, Helsinki University Hospital and University of Helsinki, Helsinki, Finland; 15 Research Unit for Pediatrics, Dermatology, Clinical Genetics, Obstetrics and Gynecology, and Medical Research Unit Oulu, Oulu University Hospital and University of Oulu, Oulu, Finland; 16 Department of General Practice and Primary Health Care, University of Helsinki, Helsinki, Finland; 17 Unit of General Practice, Helsinki University Hospital, Helsinki, Finland; 18 Folkhälsan Research Center, Helsinki, Finland; 19 Vasa Central Hospital, Vasa, Finland; 20 Research Centre of Applied and Preventive Cardiovascular Medicine, University of Turku, Turku, Finland; 21 Department of Clinical Physiology and Nuclear Medicine, Turku University Hospital, Turku, Finland; 22 Department of Epidemiology and Biostatistics, MRC-PHE Centre for Environment and Health, Imperial College London, London, UK

**Keywords:** Fetal programming, metabolic signatures, metabolomics, adiposity, fatty acids, amino acids

## Abstract

**Background:** Lower birthweight is associated with increased susceptibility to cardiometabolic diseases in adulthood, but the underlying molecular pathways are incompletely understood. We examined associations of birthweight with a comprehensive metabolic profile measured in adolescents and adults.

**Methods:** High-throughput nuclear magnetic resonance metabolomics and biochemical assays were used to quantify 87 circulating metabolic measures in seven cohorts from Finland and the UK, comprising altogether 18 288 individuals (mean age 26 years, range 15–75). Metabolic associations with birthweight were assessed by linear regression models adjusted for sex, gestational age and age at blood sampling. The metabolic associations with birthweight were compared with the corresponding associations with adult body mass index (BMI).

**Results:** Lower birthweight adjusted for gestational age was adversely associated with cardiometabolic biomarkers, including lipoprotein subclasses, fatty acids, amino acids and markers of inflammation and impaired liver function (*P* < 0.0015 for 46 measures). Associations were consistent across cohorts with different ages at metabolic profiling, but the magnitudes were weak. The pattern of metabolic deviations associated with lower birthweight resembled the metabolic signature of higher adult BMI (*R*^2^ = 0.77) assessed at the same time as the metabolic profiling. The resemblance indicated that 1 kg lower birthweight is associated with similar metabolic aberrations as caused by 0.92 units higher BMI in adulthood.

**Conclusions:** Lower birthweight adjusted for gestational age is associated with adverse biomarker aberrations across multiple metabolic pathways. Coherent metabolic signatures between lower birthweight and higher adult adiposity suggest that shared molecular pathways may potentially underpin the metabolic deviations. However, the magnitudes of metabolic associations with birthweight are modest in comparison to the effects of adiposity, implying that birthweight is only a weak indicator of the metabolic risk profile in adulthood.

Key Messages

- Lower birthweight adjusted for gestational age is adversely associated with a wide range of established and emerging circulating cardiometabolic biomarkers in adulthood, including lipoprotein subclasses and their lipids, fatty acid balance, amino acids and markers of inflammation and liver function.
- The metabolic associations are consistent across a wide age span from adolescence to retirement age, coherent for men and women, and broadly similar with and without adjustment for gestational age.
- Despite statistical significance of the metabolic associations with birthweight, the magnitudes of individual metabolic aberrations are weak for the variation in birthweight observed in general population cohorts.
- The overall metabolic association pattern with lower birthweight closely resembles the metabolic signature of higher adult adiposity. This may suggest that shared molecular pathways could underlie the fine-grained metabolic aberrations associated with both fetal growth and adiposity.
- 1-kg lower birth weight (≈ 2 SD) is associated with similar adverse metabolic effects as caused by 0.92 unit higher BMI (≈ 0.25 SD) in adulthood. These findings indicate that birthweight, as a surrogate marker for fetal growth, appears to only have modest effects on the adult metabolic risk profile in general population settings.

## Introduction

Birthweight is a marker of fetal growth rate, and lower birthweight has been associated with increased rates of ischaemic heart disease, stroke and type 2 diabetes in adulthood.^1–4^ These observations from multiple epidemiological studies have been interpreted according to the developmental origins hypothesis, which proposes that fetal adaptive responses to suboptimal nutrition *in utero* may permanently alter the fetal organ structure and metabolic homeostasis, and hereby increase susceptibility to diseases that occur later in life.^5^^,^^6^ This appears especially if fetal undernutrition is accompanied with abundant postnatal nutrition.^7^^,^^8^ The developmental origins hypothesis is supported by evidence from animal studies indicating that the fetus may adapt to an adverse intrauterine environment by slowing down growth and metabolism, which in turn has effects on adult organ size, structure and physiological function.^6^^,^^9^^,^^10^ This adaptive strategy appears to increase short-term survival, but perhaps with adverse long-term consequences on health. Also, human genetic evidence has linked fetal growth with adult metabolism and diabetes risk, yet the adverse influences of birthweight-lowering genes has been conflicting.^11^ However, despite the epidemiological evidence and animal models, the underlying molecular mechanisms linking impaired fetal growth to adult disease are poorly understood and the potential causality remains unclear.

Lifelong perturbations in causal metabolic risk factors such as elevated low-density lipoprotein (LDL) cholesterol, hypertension and type 2 diabetes, induced via fetal response to a limiting intrauterine environment, might represent pathways by which impaired fetal growth could affect cardiometabolic risk in adulthood.^2^^,^^3^ Numerous studies have shown associations between lower birthweight and adverse levels of metabolic risk factors in adulthood; these include insulin resistance and impaired glucose tolerance,^12–14^, higher blood pressure^15^ and low-grade inflammation.^16^ Also, adverse differences in cholesterol concentrations associated with lower birthweight have been reported in many studies across wide age ranges;^15^^,^^17^^,^^18^ however, meta-analyses indicate modest magnitudes of association (≈ 0.05 mmol/l higher total cholesterol per 1kg lower birthweight).^15^^,^^17^ Controversy prevails regarding the associations of birthweight with other circulating lipids, such as LDL cholesterol and triglyceride levels, and the potential relevance of such modest lipid aberrations as mediators of the adult disease risk has been questioned.^15^

The molecular effects of impaired fetal growth involve multiple metabolic pathways which extend beyond routine risk markers,^6^ and the wider influences on the systemic metabolic profile have not been assessed in large populations. Metabolomics is a powerful tool to study fine-grained molecular profiles and is therefore an attractive tool to study how birthweight is reflected in a comprehensive metabolic profile in adulthood. Nuclear magnetic resonance (NMR) metabolomics enables quantitative metabolic profiling of large blood sample collections.^19^ This methodology provides detailed lipoprotein subclass profiling, as well as quantification of fatty acids and small molecules that have recently been linked with the risk for cardiovascular disease and diabetes.^20–24^ These biomarkers could potentially serve as molecular intermediates between impaired intra-uterine growth and cardiometabolic risk. Studying the associations of birthweight with the detailed metabolic factors may therefore help to clarify the underlying mechanisms linking fetal growth with adult-onset disease and eventually help to inform how the risk could be mediated.^25^ The metabolic profiling across multiple pathways simultaneously may further provide a more comprehensive view of the systemic effects of impaired fetal growth than would be obtained by examining individual biomarkers from a single molecular pathway. However, only few metabolomics studies on birthweight have been conducted to date. These studies have been limited to a very small number of individuals, have focused on preterm birth at very low birthweight and primarily assessed associations with metabolites measured from umbilical cord blood.^26–28^ No study has previously used metabolomics to assess the role of birthweight on adult metabolic profiles in large general population settings.

To characterize metabolic signatures of lower birthweight, we used serum NMR metabolomics of 18 288 individuals from seven cohorts, which together cover individuals from adolescence to the end of working age. We further compared how the metabolic association pattern with birthweight resembles the association pattern of adiposity in adulthood for the same extensive panel of metabolic measures.

## Methods

### Study populations

The study comprised six Finnish cohorts and one cohort from the UK (Table 1): the children of the Avon Longitudinal Study of Parents and Children (ALSPAC; *n* = 2874; metabolic profiles measured from blood samples drawn at age 17);^29^ the Northern Finland Birth Cohort (NFBC) 1986 (*n* = 5579; age 16);^30^ NFBC 1966 (*n* = 5412; age 31);^31^ the Cardiovascular Risk in Young Finns Study (YFS; *n* = 2273; age 24–48);^32^ the FinnTwin studies FT12 (*n* = 767; age 21–25); FT16 (*n* = 495, age 23–30);^33^ and the Helsinki Birth Cohort Study (HBCS; *n* = 890; age 62–75).^8^ Details of the cohorts related to the present study are described in the Supplementary Methods (available as Supplementary data at *IJE* online). Birthweight and gestational age were assessed by a midwife, birth medical records or antenatal care. Gestational age was defined as a categorical variable indicating completed weeks of gestation. Out of 19 622 eligible individuals with metabolic profiling data, 18 649 had complete data on birthweight, gestational age and adult body mass index (BMI). BMI was measured in adolescence or adulthood at the same time as the blood sampling for metabolic profiling in all cohorts (henceforth denoted adult BMI). Women who were pregnant (*n* = 115) and individuals on lipid-lowering medication (*n* = 246) at the time of metabolic profiling were excluded, leaving 18 288 individuals for the present analysis. All study participants provided informed consent, and study protocols were approved by the local ethics committees.

**Table 1.** Characteristics of the seven cohorts

Characteristics	NFBC 1986	ALSPAC Children	Finn-Twin	Finn-Twin	NFBC 1966	Young Finns Study	Helsinki Birth Cohort Study
			FT12	FT16			
Number of individuals	5579	2874	767	495	5412	2273	890
Men [%]	50.4	48.4	41.2	51.1	49.9	44.7	43.9
Age at blood sampling [years]	16.1 (0.4)	17.8 (0.4)	22.4 (0.6)	26.2 (1.3)	31.2 (0.4)	39.2 (6.1)	66.3 (2.9)
Birthweight [g]	3562 (541)	3426 (546)	2703 (507)	2673 (495)	3498 (523)	3508 (543)	3432 (470)
Gestational age [week]	39.4 (1.8)	39.4 (1.6)	36.9 (2.2)	36.9 (2.5)	40.0 (1.9)	38.6 (1.2)	40.0 (1.5)
Adult body mass index [kg/m^2^]	21.2 (3.4)	23.0 (3.9)	23.3 (3.9)	23.8 (4.0)	25.6 (4.1)	26.3 (4.9)	27.0 (4.1)
Systolic blood pressure [mm Hg]	116 (13)	115 (10)	–	–	125 (13)	119 (14)	143 (19)
Total-C [mmol/l]	4.3 (0.8)	3.8 (0.7)	4.7 (0.9)	5.0 (0.9)	5.0 (1.0)	5.1 (0.9)	6.1 (1.0)
HDL-C [mmol/l]	1.4 (0.3)	1.4 (0.2)	1.8 (0.4)	1.8 (0.4)	1.5 (0.4)	1.3 (0.3)	1.7 (0.4)
Triglycerides [mmol/l]	0.7	0.8	0.9	1.2	1.0	1.1	1.2
	[0.6-1.0]	[0.7-1.0]	[0.7-1.2]	[0.9-1.62]	[0.7-1.4]	[0.8-1.5]	[0.9-1.7]
Glucose [mmol/l]	5.2	5.0	4.6	4.7	5.0	5.3	5.5
	[4.9-5.4]	[4.7-5.3]	[4.3-4.8]	[4.3-5.2]	[4.7-5.3]	[4.9-5.6]	[5.1-5.8]
Insulin [IU/l]	9.6	6.8	–	–	7.5	7.3	7.4
	[7.4-12.3]	[4.9-9.3]			[6.2-9.4]	[4.4-11.2]	[5.2-11.2]

Values are mean (SD) and median [interquartile range] for normally distributed and positively skewed variables, respectively.

### Lipid and metabolite quantification

Fasting blood samples were collected as part of the clinical examinations in adolescence and in adulthood and stored as serum or EDTA plasma at -80 °C for subsequent biomarker profiling, as detailed in Supplementary Methods. Altogether 87 metabolic measures were analysed for the present study. A high-throughput NMR metabolomics platform was used for the quantification of 77 metabolic measures.^19^ This metabolomics platform provides simultaneous quantification of routine lipids and lipid concentrations of 14 lipoprotein subclasses and major subfractions, and further quantifies abundant fatty acids, amino acids, ketone bodies and gluconeogenesis-related metabolites in absolute concentration units (Table S1, available as Supplementary data at *IJE* online). The metabolic profiling therefore includes both routine risk markers and novel metabolic biomarkers that have not previously been examined in relation to birthweight. The NMR metabolomics platform has been extensively applied for biomarker profiling in epidemiological studies^20^^,^^21^^,^^23–25^^,^^34^^,^^35^ and details of the experimentation have been described elsewhere.^19^^,^^36^ In addition to the NMR metabolomics measures, 10 metabolic markers related to inflammation, liver function and hormone balance, assayed in two or more of the cohorts, were analysed as part of the comprehensive metabolic profile (Supplementary Methods).^25^ Mean (standard deviation; SD) concentrations of the metabolic measures in each cohort are listed in Table S2, available as Supplementary data at *IJE* online.

### Statistical analyses

Metabolic measures with skewed distributions (skewness > 2) were normalized by log-transformation before analysis. Linear regression models for each metabolite were tested with birthweight as the explanatory variable and the metabolite concentration as an outcome. Associations were adjusted for sex, age at blood sampling and gestational age. Adjusting birthweight for gestational age broadly means that the birthweight variable may be interpreted in terms of growth rate. Results were analysed separately for the seven cohorts and combined using fixed effect inverse-variance weighted meta-analysis after verifying the consistency across the seven cohorts. Association magnitudes are quantified in SD units of metabolite concentration per 1kg lower birthweight (≈ 2 SD). Due to the correlated nature of the metabolic measures, > 95% of the variation in the 87 measures was explained by at most 34 principal components in each cohort. Multiple testing correction therefore accounted for 34 independent tests using the Bonferroni method, resulting in *P* < 0.0015 being denoted statistically significant.

The pattern of metabolic associations with birthweight was compared with the corresponding cross-sectional metabolic associations with adult BMI, using the same approach as used for birthweight but without adjustment for gestational age. The overall correspondence between the metabolic association patterns of birthweight and adult BMI were summarized using the *R*^2^ and slope of the linear fit.^25^^,^^35^

To examine whether there was evidence for curvilinear associations, we assessed the shape of the associations using local quadratic regression fitting, with each smoothing function evaluated at 25 points through the range of birthweight. Absolute concentrations of each metabolic measure were first regressed for age and sex, and the resulting residuals were pooled and re-scaled to absolute units before fitting.^35^

## Results

The study comprised 18 288 adolescents and adults from five general population cohorts and two twin cohorts from Finland and the UK (Table 1). Distributions of birthweight for each cohort are illustrated in Figure S1, available as Supplementary data at *IJE* online. Only 1% of the singleton participants had a birthweight of < 2 kg. Birthweight was correlated with adult BMI (*r* = 0.09) in a broadly linear manner for most of the cohorts (Figure S2, available as Supplementary data at *IJE* online).

### Lipoprotein measures

**Figure 1.**
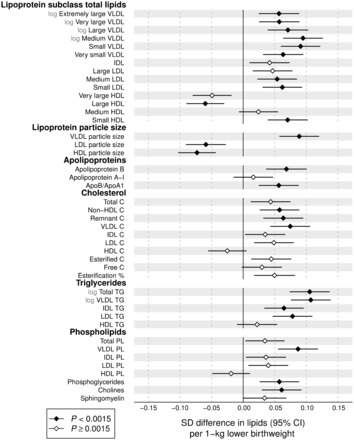
Birthweight associations with adult concentrations of lipoprotein lipids. The associations were adjusted for sex, gestational age and age at blood sampling, and meta-analysed for 18 288 individuals from seven cohorts. Association magnitudes are 1 SD lipid concentration per 1 kg lower birthweight. Error bars denote 95% confidence intervals. Filled diamonds indicate *P* < 0.0015. Association magnitudes in absolute concentration units and *P*-values are listed in Table S1, and results for individual cohorts are shown in Figure S5, both available at *IJE* online.

Associations of birthweight with 43 lipoprotein measures are shown in Figure 1. In the meta-analysis, birthweight was robustly associated with numerous lipoprotein measures (*P* < 0.0015 for 25 measures). Whereas the associations of routine cholesterol measures were of modest magnitudes, somewhat stronger associations were observed for many of the more detailed lipid measures. Lower birthweight was associated with higher circulating apolipoprotein B (apoB) and total lipid concentrations in the apoB-carrying particles [very-low-density lipoprotein (VLDL), intermediate-density lipoprotein (IDL) and LDL]. The strongest associations with lipoprotein subclasses were observed for lipids in medium and small VLDL particles. Associations were weaker for lipids in IDL and LDL particles, albeit with stronger association magnitude for lipids in small LDL. Associations were more heterogeneous for lipids in high-density lipoprotein (HDL) particles: lower birthweight was associated with lower concentrations of circulating lipids in large HDL particles, but with higher concentrations of those in small HDL (i.e. in the same direction as for the apoB-carrying lipoproteins). Birthweight was also robustly associated with the average size of the lipoprotein particles, with increased VLDL size and decreased LDL and HDL size related to lower birthweight. Within a given lipoprotein class, associations with birthweight tended to be stronger for triglycerides than for cholesterol and phospholipid levels.

To enable comparison of birthweight associations across the metabolic measures, all association magnitudes are scaled to SD units of metabolite concentration per 1kg lower birthweight. The corresponding associations in absolute units, e.g., mmol/l/kg, are listed in Table S1. For instance, total triglyceride concentration was among the measures most strongly associated with birthweight, with each 1kg lower birthweight being associated with 0.04 mmol/l higher serum triglyceride concentration.

### Fatty acids

**Figure 2.**
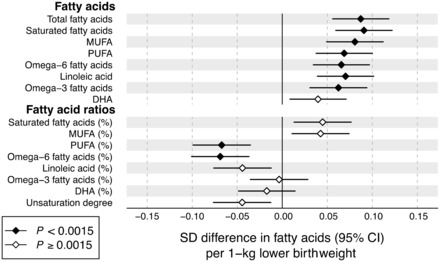
Birthweight associations with adult fatty acid levels. The associations were adjusted for sex, gestational age and age at blood sampling, and meta-analysed for 18 288 individuals from seven cohorts. Association magnitudes are in units of 1 SD fatty acid measure per 1 kg lower birthweight. Fatty acid ratios are relative to the total fatty acid concentration. Error bars denote 95% confidence intervals. Filled diamonds indicate *P* < 0.0015. Results for individual cohorts are shown in Figure S5, available at *IJE* online. MUFA, mono-unsaturated fatty acids; PUFA, polyunsaturated fatty acids; DHA, docosahexaenoic acid.

Associations of birthweight with 16 fatty acid measures are shown in Figure 2. Lower birthweight was robustly associated with higher absolute concentration of all fatty acids assayed except docosahexaenoic acid. The strongest associations were observed for total, saturated and monounsaturated fatty acids (MUFA), which displayed association magnitudes comparable to that of apoB. Somewhat weaker associations were observed for omega-6 and omega-3 fatty acids. For the fatty acid ratios, the proportion of saturated fatty acids and MUFAs tended to be higher among individuals with lower birthweight, whereas the proportion of omega-6 fatty acids was lower.

### Non-lipid metabolic measures

**Figure 3.**
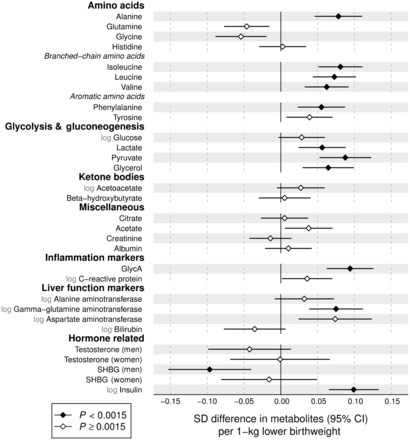
Birthweight associations with adult metabolite and hormonal concentrations. The associations were adjusted for sex, gestational age and age at blood sampling, and meta-analysed for 18 288 individuals from seven cohorts. Association magnitudes are in units of 1 SD metabolite concentration per 1 kg lower birthweight. Error bars denote 95% confidence intervals. Filled diamonds indicate *P* < 0.0015. Results for individual cohorts are shown in Figure S5, available at *IJE* online. GlycA, glycoprotein acetyls; SHBG, sex-hormone binding globulin.

Associations of birthweight with 28 non-lipid metabolites and other metabolic measures are shown in Figure 3. Lower birthweight was associated with higher concentrations of alanine, branched-chain and aromatic amino acids. These amino acid associations with birthweight were of comparable magnitude to that of apoB. Lower birthweight was not robustly associated with glucose, but other gluconeogenesis-related metabolites displayed stronger associations. Lower birthweight was also robustly associated with higher levels of insulin and certain markers for low-grade inflammation and impaired liver function.

### Resemblance between metabolic signatures of birthweight and adult BMI

**Figure 4.**
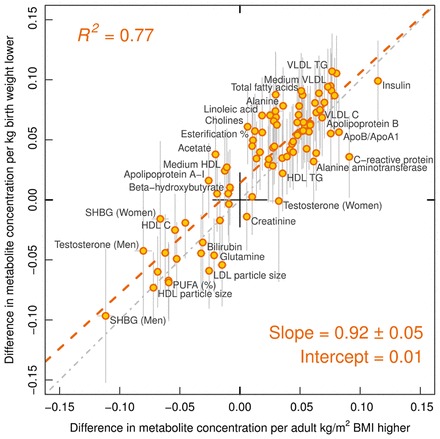
Resemblance between metabolic association patterns related to lower birthweight and higher adulthood BMI (assessed at the same time as blood sampling for metabolic profiling). The metabolic associations were assessed for the same 18 288 individuals. The red dashed line denotes the linear fit between metabolic associations with lower birthweight and higher BMI. The slope indicates that 1kg lower birthweight is on average associated with metabolic deviations similar to those linked with 0.92 kg/m^2^ higher BMI in adulthood.

The overall pattern of metabolic associations with lower birthweight was reminiscent of the metabolic association pattern with adiposity assessed at the same time as blood sampling (Figure S3, available as Supplementary data at *IJE* online).^25^^,^^37^^,^^38^ We therefore compared the metabolic associations of birthweight to the corresponding metabolic associations of adult BMI (Figure 4). The resemblance between the metabolic association patterns was high, as indicated by the goodness-of-fit being *R*^2^ = 0.77. The slope denotes that 1kg lower birthweight is associated with similar magnitudes of metabolic aberrations as those linked with 0.92 higher adult BMI units (kg/m^2^). A similar resemblance with the association pattern with lower birthweight was observed for higher adult weight (*R*^2^ = 0.75), indicating that metabolic associations with 1kg lower birthweight were similar to those linked with 3.1kg higher adult weight. In contrast, the pattern of metabolic associations with adult height was considerably different (Figure S4, available as Supplementary data at *IJE* online; *R*^2^ = 0.17).

### Consistency and sensitivity analyses

Despite differences in age at blood sampling, the associations of birthweight with the metabolic measures were generally consistent across the seven cohorts and displayed little evidence of heterogeneity (Figure S5, available as Supplementary data at *IJE* online). The results were also generally similar and statistically consistent for men and women (Figure S6, available as Supplementary data at *IJE* online). The association magnitudes were also similar (on average 5% weaker) if omitting participants within the lowest percentile (birthweight < 2.0 kg) and highest percentile (birthweight > 4.7 kg). The metabolic associations were slightly weaker (on average 8%) if omitting gestational age as a covariate (Figure S7, available as Supplementary data at *IJE* online). However, gestational age modelled as the primary exposure was essentially not associated with metabolic aberrations in adulthood (Figure S7), indicating that growth rate rather than length of gestation is important in relation to metabolic deviations.^39^ The metabolic associations with birthweight followed a similar pattern if adjusting for adult BMI (assessed at the same time as the blood sampling); however, this adjustment increased the magnitude of association by 58% on average (Figure S8, available as Supplementary data at *IJE* online). The continuous shapes of the metabolic associations with birthweight are illustrated in Figure S9, available as Supplementary data at *IJE* online*.* Most associations were approximately linear across the birthweight distribution, justifying the use of linear regression modelling.

## Discussion

In this meta-analysis of 18 288 individuals, lower birthweight (adjusted for gestational age) was adversely associated with numerous blood-based biomarkers across the comprehensive metabolic profile, including lipoprotein subclass measures, fatty acid composition, amino acids and markers of inflammation and liver function. The metabolic associations were all in a direction of higher risk for diabetes and cardiovascular disease, both for established risk factors and emerging biomarkers. The associations were coherent across seven cohorts with a wide age range at metabolic profiling, from adolescence through adulthood, suggesting that these metabolic aberrations are lifelong. These findings are coherent with the developmental origins hypothesis, proposing long-term alterations in metabolic homeostasis.^6^ However, although many of the associations were statistically robust in this large study sample, the small association magnitudes indicate that the influences of birthweight on systemic metabolism are modest.

The wide palette of the metabolic deviations associated with lower birthweight have previously been linked with increased risk for cardiometabolic diseases.^19–24^ Among the lipoprotein measures adversely associated with lower birthweight were both cholesterol-rich LDL particles and triglyceride-rich VLDL particles. Genetic evidence suggests that both these types of apoB-carrying lipoproteins are causally related to ischaemic heart disease.^40^ Associations with routine cholesterol measures were modest, in line with previous meta-analyses that have questioned the relevance of associations between birthweight and circulating lipids.^15^^,^^17^^,^^41^ Although the lipoprotein subclass profiling indicated somewhat stronger and statistically robust associations with several more detailed lipid measures, the associations were nevertheless in line with the overall weak metabolic aberrations.

Increased circulating levels of omega-3 fatty acids have been associated with lower risk for future cardiovascular disease events.^20^ Dietary supplementation of omega-3 fatty acids has been suggested as intervention for people born with impaired fetal growth,^42^ but the serum levels of omega-3 have not been robustly linked with birthweight. Here, the association of birthweight was flat for the proportion of omega-3 fatty acids relative to total fatty acids. However, the proportion of omega-6 fatty acids—also inversely associated with the risk for cardiovascular disease^20^ and diabetes^23^—was decreased in relation to lower birthweight. The small magnitudes of the fatty acid perturbations are unlikely to substantially mediate the associations between lower birthweight and increased susceptibility to cardiometabolic diseases.

Recent metabolomics studies have shown that amino acids and many other circulating metabolites are predictive of the risk for diabetes, cardiovascular disease and all-cause mortality.^20–22^^,^^24^^,^^43^ For instance, elevated circulating levels of branched-chain and aromatic amino acids have been robustly associated with insulin resistance, hyperglycaemia and diabetes risk in a number of studies.^22^^,^^24^^,^^43^ Aromatic amino acids have also been shown to be predictors of cardiovascular event risk, even more strongly than LDL cholesterol.^20^ All these amino acids were found to be elevated for lower birthweight, that is in the direction consistent with higher metabolic risk. Lower birthweight was also related to higher concentrations of markers for impaired liver function, insulin resistance and chronic inflammation. These metabolic markers are also predictive for the risk of a broad span of chronic diseases and all-cause mortality.^21^^,^^44^^,^^45^ Importantly, although the individual biomarker associations with birthweight are all modest, the combined metabolic aberrations may in concert potentially contribute to mediate the relation between birthweight and cardiometabolic disease risk.

Elevated BMI has recently been shown to have a causal metabolic signature across biomarkers from multiple pathways.^25^ This metabolic signature of adiposity is highly reminiscent of the pattern of metabolic associations here linked with lower birthweight. The similarity in the detailed association patterns illustrates how comprehensive metabolic profiling may help to pinpoint molecular connections between the metabolic effects of different risk factors. The resemblance between the metabolic signatures allows summary of the metabolic aberrations linked with birthweight in relation to the effects of adiposity: 1kg lower birthweight in the general population is associated with metabolic deviations similar to those caused by 0.92 kg/m^2^ higher BMI in adulthood. The consistency with the metabolic association pattern for birthweight was driven by adult weight rather than height, suggesting that the underlying mechanisms are not primarily related to stature. A 1 kg of difference in birthweight is substantial, corresponding to almost 2 SDs. A similar variance in adulthood BMI (i.e. 2 SD greater adult BMI) is associated with 8-fold stronger metabolic deviations, indicating much more pronounced metabolic perturbations associated with higher adult adiposity than those linked with lower birthweight.

The prominent correspondence between the metabolic signatures of lower birthweight and elevated BMI leads us to speculate that impaired fetal growth and adiposity may have shared molecular pathways underlying the fine-grained metabolic aberrations. As a corollary of this hypothesis, we anticipate that the causal effects of BMI also on other molecular markers^46^ and physiological factors can be used to predict the anticipated association of birthweight with these same outcomes. For instance, genetic evidence indicates that the lifelong causal effect of BMI on systolic blood pressure is 0.9 mmHg per higher BMI unit;^25^^,^^47^ based on the above hypothesis, we extrapolate that the association of birthweight with systolic blood pressure would be 0.9 × 0.92 ≈ 0.8 mmHg/kg lower birthweight. This is broadly consistent with the association observed in meta-analysis.^37^^,^^38^ Since our results indicate that the metabolic perturbations associated with birthweight are present across the life course, it is important that such extrapolation of birthweight associations with other risk markers is based on lifelong effects of BMI, for example from genetic estimates. Based on this principle, it is also possible to estimate the association of birthweight with cardiometabolic disease risk by comparison to the risk effects caused by BMI. Accordingly, 0.92 BMI unit is causally associated with ≈ 10% higher risk for ischaemic heart disease,^48^^,^^49^ which is consistent with meta-analysis results on the risk magnitude for ischaemic heart disease per 1 kg lower birthweight.^2^ Similarly, 0.92 BMI unit is causally associated with 24–33% higher risk for type 2 diabetes,^47^^,^^50^ which again is consistent with meta-analysis estimates of the diabetes risk per 1 kg lower birthweight.^3^ These results seem to suggest that the comprehensive metabolic effects corresponding to as little as 0.92 BMI units, affecting over the life course, may be sufficient to explain the association of birthweight with cardiometabolic disease risk in adulthood. However, we acknowledge that the specific mechanisms linking birthweight and adiposity to cardiometabolic outcomes may be different despite the shared metabolic signatures.

It is of note that although birthweight was ≈ 700 g lower in the twin cohorts than in singletons, the metabolite concentrations and the pattern of metabolic associations with birthweight were generally similar to the other cohorts (Table S2 and Figure S5). This is consistent with no difference in diabetes prevalence or overall mortality among twin individuals compared with singletons,^51^^,^^52^ supporting our conclusion that lower birthweight per se has a minor long-term impact on the systemic metabolic risk profile. However, direct comparison of potential small metabolic differences due to lower birthweight in twins is not feasible in this study due to confounding differences in cohort characteristics as well as variations between cohorts in pre-analytic sample handling, storage duration and type of blood specimen (serum vs plasma). The metabolic associations with birthweight were stronger (on average 58%) if adjusting for adult BMI, in line with previous studies on metabolic risk factors,^3^^,^^15^^,^^17^^,^^37^ suggesting that birthweight in relation to current weight is more relevant for adulthood metabolic aberrations than birthweight alone. The adjustment for adult BMI has been considered inappropriate due to the correlation of BMI with both birthweight and metabolite levels.^37^^,^^53^ However, the BMI-adjusted results may be interpreted as a measure of change in size between birth and adulthood,^53^^,^^54^ and the stronger metabolic associations observed here accordingly indicate a contributing role of postnatal growth. Further studies on the comprehensive metabolic effects of growth in infancy may clarify the role of compensatory growth and other proposed interactions with birthweight on the metabolic profile in adulthood.^8^^,^^54^

Strengths of this study include the large sample size, comprising seven cohorts with quantitative metabolomics data. Birthweight was obtained from birth medical records for 88% of the study population, which minimizes bias from self-reporting, and data on gestational age allowed accounting for lower birthweight caused by prematurity. The broadly coherent results across cohorts of a wide age range provided a view of the life-course effects of birthweight. However, the general population nature of the cohorts analysed prevents us from making conclusions regarding the specific metabolic effects of infants born preterm, or those with severe fetal growth restriction.^28^ Birthweight has limitations as a marker of impaired fetal growth; however, it is the surrogate most widely reported in large population cohorts, and it has a high correlation with other markers of size at birth.^17^ We acknowledge that we cannot assume that impaired fetal growth is causal for the adulthood metabolic aberrations observed. For example, genetic analyses support a causal role for maternal smoking in pregnancy and higher blood pressure resulting in lower infant birthweight, which could potentially contribute to explain the weak inverse associations with metabolic risk markers observed here.^55^^,^^56^ Given the predominantly young age of the study participants, we were not able to test whether the metabolic aberrations related to birthweight could mediate the relationship to cardiometabolic disease outcomes. Other metabolomics technologies may eventually provide further insights into the intricate metabolic effects of fetal growth. However, the present study demonstrates that large sample size is required to robustly assess the weak associations. Finally, it is important to recognize that information on antenatal nutrition and other factors that might underlie the relationships of birthweight with metabolic outcomes might highlight associations of a larger magnitude or potentially greater importance than suggested by our results.

In conclusion, comprehensive metabolic profiling of large cohorts identified associations between lower birthweight (adjusted for gestational age) and adverse circulating levels of a wide panel of circulating cardiometabolic risk markers in adulthood. The overall metabolic signature of lower birthweight closely resembled the metabolic effects of higher BMI, suggesting that shared molecular pathways may underpin the perturbed metabolic profile related to both fetal growth and adiposity. Nevertheless, the aberrations were of modest magnitude, with similar metabolic perturbations related to 1 kg lower birthweight as those caused by lifelong effects of ≈ 3 kg higher body weight in adulthood. These results indicate that birthweight is only a weak indicator of the metabolic risk profile in adulthood.

## Funding

This study was supported by the Strategic Research Funding from the University of Oulu, Finland, the Sigrid Juselius Foundation, the Novo Nordisk Foundation, the Yrjö Jahnsson Foundation, the Finnish Diabetes Research Foundation, the Finnish Medical Foundation, the Paulo Foundation, Biocenter Oulu, Finland, and the UK Medical Research Council via the University of Bristol Integrative Epidemiology Unit (IEU; MC_UU_12013/1 and MC_UU_12013/5). The Cardiovascular Risk in Young Finns Study is supported by the Academy of Finland (grants 286284, 134309, 126925, 121584, 124282, 129378, 117787 and 41071), Finnish Foundation for Cardiovascular Research, Oulu, Helsinki, Kuopio, Tampere, and Turku University Central Hospital Medical Funds, the Paavo Nurmi Foundation, the Juho Vainio Foundation, the Finnish Cultural Foundation and the Finnish Funding Agency for Technology and Innovation. The Northern Finland Birth Cohorts of 1966 and 1986 have received financial support from Academy of Finland, University Hospital Oulu, Biocenter Oulu, University of Oulu, the European Commission (EURO-BLCS, Framework 5 Award QLG1-CT-2000-01643, ENGAGE project and grant agreement HEALTH-F4-2007-201413, EurHEALTHAgeing (277849), European Regional Developmental Fund), EU H2020-PHC-2014 (grant no. 633595), DynaHEALTH, NHLBI grant 5R01HL087679-02 through the STAMPEED programme (1RL1MH083268-01), NIH/NIMH (5R01MH63706:02), Stanley Foundation, the UK Medical Research Council and Wellcome Trust. The UK Medical Research Council and Wellcome Trust (grant: 102215/2/13/2) and the University of Bristol provide core support for ALSPAC. The contribution of D.A.L to this study is supported by grants from the US National Institute of Health (R01 DK10324), European Research Council (ObesityDevelop: grant no. 669545) and UK National Institute for Health Research (NF-SI-0166-10196). F.D., D.A.L., G.D.S. and M.A.K. work in a Unit that is supported by the University of Bristol and UK Medical Research Council (MC_UU_12013/1 and MC_UU_12013/5). The FinnTwin-12 and FinnTwin-16 studies have received support from the National Institute on Alcohol Abuse and Alcoholism (R37-AA12502, R01-AA09203 and K05-AA00145). The views expressed in this paper are those of the authors and not necessarily any funding body.

**Conflict of interest:** P.W., A.J.K., P.S. and M.A.K. are shareholders of Brainshake Ltd, a company offering NMR-based metabolic profiling (www.brainshake.fi). P.W., T.T., M.T., P.S. and A.J.K. report employment relation with Brainshake Ltd. D.A.L. has received funding for biomarker research, unrelated to this paper, from Roche Diagnostics and Ferring Pharmaceuticals. K.A. is currently employed by GSK. No other authors reported disclosures.

## Supplementary Material

Supplementary Data
