# Supplementary material for: Metabolic signatures of birthweight in 18 288 adolescents and adults
Source: Int J Epidemiol. 2016 Nov 7;45(5):1539–50. doi: 10.1093/ije/dyw255 (PMC5100627; doi:10.1093/ije/dyw255)
Supplement: Supplementary Data [file dyw255_supplementary_data.zip › ije-2016-04-0469-File002.docx]

**Supplementary Data**

**Würtz et al, Metabolic signatures of birth weight in 18 288 adolescents and adults.**

**Supplementary Methods: Study populations.**

**Table S1. Mean (SD) metabolic concentrations, and associations with birth weight in absolute concentration units.**

**Table S2. Mean (SD) concentrations of metabolites in each cohort.**

**Figure S1. Birth weight distribution in each cohort.**

**Figure S2. Adult body mass index as a function of birth weight in each cohort.**

**Figure S3. Metabolic associations with adulthood body mass index.**

**Figure S4. Metabolic associations with adulthood height.**

**Figure S5. Metabolic associations with birth weight in each cohort.**

**Figure S6. Metabolic associations with birth weight for men and women.**

**Figure S7. Metabolic associations with birth weight without adjustment for gestational age and gestational age as predictor of adulthood metabolic aberrations.**

**Figure S8. Metabolic associations with birth weight adjusted for adult BMI.**

**Figure S9. Curvilinear shapes of metabolic associations with birth weight.**

**Supplementary Methods: Study populations**

*The Avon Longitudinal Study of Parents and Children (ALSPAC) Children’s Cohort*

The Avon Longitudinal Study of Children and Parents (ALSPAC) was established to understand how genetic and environmental characteristics influence health and development in parents and children ([www.bristol.ac.uk/alspac](http://www.bristol.ac.uk/alspac/)). Recruitment sought to enrol pregnant women in the Bristol area of the United Kingdom during 1990–1992. The children from 14 541 pregnancies were recruited in 1990–1992.^1^ Metabolic profiling data analysed for the present study was conducted from fasting plasma samples collected during the fourth teen focus survey when participants were 17 years of age (2007–2009). At this follow-up survey there were 2874 participants with metabolic profiling data available and information on birth weight, gestational age and BMI at blood sampling. In addition to NMR-based metabolic profiling, plasma levels of C‑reactive protein and insulin were measured by standard clinical chemistry assays. The study was approved by the ethics committees of the University of Bristol, UK, and all study participants provided written informed consent.

*Northern Finland Birth Cohorts of 1986 (NFBC1986)*

The Northern Finland Birth Cohorts (NFBC) of 1986 and 1966 were initiated to study factors affecting preterm birth and subsequent morbidity in the two northernmost provinces in Finland (www.oulu.fi/NFBC). For NFBC1986, the number of deliveries in the birth cohort was 9362, which was 99% of all the deliveries taking place in the area during the target period (July 1985–June 1986).^2,3,4^ Data collection in 2001–2002 included clinical examination and serum sampling at age 15–16 for 6621 adolescents; attendees in the 16-year field study (71% of invited participants) were representative of the original cohort.^4^ Metabolic profiling data from this timepoint are used in the present study. In total, 5579 adolescents had a comprehensive metabolic profile measured and information on birth weight, gestational age and BMI at blood sampling (age 16). Of these, 95% were based on serum samples drawn after overnight fasting. Informed written consent was obtained from all participants. In addition to NMR-based metabolic profiling, plasma levels of the following protein biomarkers were measured by standard clinical chemistry assays and analysed in the present study: C‑reactive protein, alanine aminotransferase, gamma-glutamyl aminotransferase, aspartate aminotransferase, bilirubin and insulin.^5^ Testosterone and sex-hormone binding globulin were measured by mass spectrometry.^6^ The research protocols were approved by the Ethics Committee of Northern Ostrobotnia Hospital District, Finland.

*Northern Finland Birth Cohorts of 1966 (NFBC1966)*

The NFBC1966 included 12 058 children born alive into the cohort, comprising 96% of all births during 1966 in the region.^7^ Data collection in 1997 included clinical examination and serum sampling at age 31 for 6007 individuals. Attendees in the 31-year field study (≈72% of the eligible population invited) were representative of the original cohort.^7^ Metabolic profiling data from this time point are used for the present study. In total, 5412 persons had comprehensive metabolic profile data and information available on birth weight, gestational age and BMI at blood sampling (age 31). The serum samples were collected after overnight fast for 96% of the study participants. In addition to NMR-based metabolic profiling, plasma levels of the following protein biomarkers were measured by standard clinical chemistry assays and analysed in the present study: C‑reactive protein, alanine aminotransferase, gamma-glutamyl aminotransferase, bilirubin and insulin.^5^ Testosterone and sex-hormone binding globulin were measured by mass spectrometry.^6^ Pregnant women were excluded from the analyses. Informed written consent was obtained from all participants. The research protocols were approved by the Ethics Committee of University of Oulu and the Ethics Committee of Northern Ostrobotnia Hospital District, Finland.

*The FinnTwin12 (FT12)*

The FinnTwin12 study is a longitudinal study of five consecutive birth cohorts (1983–1987) of Finnish adolescent twins focusing on behavioural development and health habits ([www.twinstudy.helsinki.fi](http://www.twinstudy.helsinki.fi)). Around 2600 families with twins initially enrolled during adolescence, and have repeatedly been assessed by self-reported questionnaires.^8^ During the fourth wave of the data collection, a subsample of these young adult twins visited the study clinic where height and weight were measured and fasting blood samples for NMR metabolomics were drawn.^9^ Birth weight and gestational age were reported by the twins’ mother at baseline.^10^ Metabolic data were available for 775 individuals, among whom 767 had complete data on birth weight, gestational age and adulthood BMI. Data collection and analysis were approved by the ethics committee of the Department of Public Health of the University of Helsinki and the Institutional Review Board of Indiana University, USA. Written informed consent was obtained from all participants or their parents.

*The FinnTwin16 Study (FT16)*

The FinnTwin16 study is a longitudinal study of twins born between 1975 and 1979 and their parents and siblings with a total of 5594 participants in the baseline.^8^ The focus of the longitudinal study is the behavioural development and health habits of Finnish twins initially enrolled during adolescence ([www.twinstudy.helsinki.fi](http://www.twinstudy.helsinki.fi)). Three adolescent questionnaires were administered at ages 16, 17 and 18.5 years. During the fourth wave of the data collection, a subsample of these young adult twins visited the study clinic where height and weight were measured and fasting blood samples for NMR metabolomics were drawn. Participants were allowed to have a light breakfast prior to blood sampling (e.g. a cup of coffee or tea, bread, fruits etc.), but not anything very greasy (e.g. bacon and eggs).^9^ Birth weight and gestational age were reported by the twins’ mother at baseline.^10^ Metabolic data were available for 560, among whom 495 had complete data on birth weight, gestational age and adulthood BMI. Data collection and analysis were approved by the ethics committee of the Department of Public Health of the University of Helsinki and the Institutional Review Board of Indiana University, USA. Written informed consent was obtained from all participants or their parents.

*The Cardiovascular Risk in Young Finns Study*

The Cardiovascular Risk in Young Finns Study (YFS) was designed to study associations of childhood risk factors to cardiovascular disease in adulthood (youngfinnsstudy.utu.fi).^11,12^ The baseline study in 1980 included 3596 children and adolescents aged 3–18. In contrast to all the other cohorts, birth weight was not assessed directly from hospital recordings, but rather at follow-up visits in 1983 and 1986, where the participants together with their parents completed a detailed questionnaire, including information on birth weight and gestation. Birth characteristics were confirmed from participants’ records from well-baby clinics. In contrast to all the other cohorts analysed in this study, information on gestational age was only available if the delivery was prior to week 39.^13^ Metabolic profiling data from the 2011 survey are used in the present study (n=1671). In addition, 607 individuals with metabolic profiling data who only attended the 2001 or 2007 surveys were included in analyses. In total, the analyses included 2273 individuals with an overnight fasting metabolic profile and information on birth weight, gestational age and adulthood BMI. These individuals were representative of the original cohort. In addition to NMR-based metabolic profiling, plasma levels of the following protein biomarkers were measured by standard clinical chemistry assays and analysed in the present study: C‑reactive protein, alanine aminotransferase, gamma-glutamyl aminotransferase, and insulin.^5^ Pregnant women and individuals on lipid-lowering medication were omitted from the analyses. All participants gave written informed consent. The study was approved by the ethics committees of each of the five participating medical university study sites in Finland.

*Helsinki Birth Cohort Study (HBCS)*

The HBCS 1934–1944 included 13 345 subjects born at Helsinki University Central Hospital or at the Helsinki City Maternity Hospital, Finland ([research.med.helsinki.fi/obesity/Eriksson/](http://research.med.helsinki.fi/obesity/Eriksson/)).^14^ Information on prenatal and childhood growth was collected from hospital birth records, child welfare clinics, and school health care records. These records included information on health and growth during childhood as well as socioeconomic factors. The hospital birth records included data on birth weight, gestational age, and maternal characteristics. A clinical examination during years 2006-2008 (at 62–74 years of age) included 1083 individuals with anthropometric data and fasting blood samples collected. Among these, NMR metabolomics data were measured for 1078 participants with complete data on birth weight, gestational age and BMI at blood sampling. In addition to NMR-based metabolic profiling, plasma levels of insulin were measured by standard clinical chemistry assays. Participants using lipid-lowering medication (n=188) were excluded from the analyses, leaving 890 individuals to be included in the present analyses. All participants signed informed written consent and the study was approved by the Ethics Committees of National Public Health Institute and the Helsinki and Uusimaa Hospital District, Finland.

**References**

1. Boyd et al. Cohort Profile: The ‘Children of the 90s’—the index offspring of the Avon Longitudinal Study of Parents and Children. *Int J Epidemiol* 2013;**42**:111–27.
2. Järvelin MR, Hartikainen-Sorri AL, Rantakallio P. Labour induction policy in hospitals of different levels of specialisation. *Br J Obstet Gynaecol* 1993;**100**:310–315.
3. Kantomaa MT, Stamatakis E, Kankaanpää A, et al. Physical activity and obesity mediate the association between childhood motor function and adolescents' academic achievement. Proc Natl Acad Sci U S A 2013;**110**:1917–1922.
4. Veltsista A, Laitinen J, Sovio U, Roma E, Järvelin MR, Bakoula C. Relationship between eating behavior, breakfast consumption, and obesity among Finnish and Greek adolescents. *J Nutr Educ Behav* 2010;**42**:417–421.
5. Würtz P, Wang Q, Kangas AJ, et al. Metabolic signatures of adiposity in young adults: Mendelian randomization analysis and effects of weight change. *PLoS Med* 2014;**11**:e1001765.
6. Wang Q, Kangas AJK, Soininen P, et al. Sex hormone-binding globulin associations with circulating lipids and metabolites and the risk for type 2 diabetes: observational and causal effect estimates. *Int J Epidemiol* 2015;**44**:623–37.
7. Järvelin MR, Sovio U, King V, et al. Early life factors and blood pressure at age 31 years in the 1966 northern Finland birth cohort. *Hypertension* 2004;**44**:838–46.
8. Jelenkovic A, Bogl LH, Rose RJ, et al. Association between serum fatty acids and lipoprotein subclass profile in healthy young adults: exploring common genetic and environmental factors. *Atherosclerosis* 2014;**233**:394–402.
9. Kaprio J. Twin studies in Finland 2006. *Twin Res Hum Genet* 2006;**9**:772–777.
10. Heikkilä K, Vuoksimaa E, Saari-Kemppainen A, et al. Higher prevalence of left-handedness in twins? not after controlling birth time confounders. *Twin Res Hum Genet* 2015;18:526-32.
11. Raitakari OT, Juonala M, Rönnemaa T, et al. Cohort profile: The cardiovascular risk in young Finns study. *Int J Epidemiol* 2008;**37**:1220–1226.
12. Nuotio J, Oikonen M, Magnussen CG, et al. Cardiovascular risk factors in 2011 and secular trends since 2007: the Cardiovascular Risk in Young Finns Study. *Scand J Public Health* 2014;**42**:563–71.
13. Skilton MR, Viikari JSA, Juonala M, et al. Fetal growth and preterm birth influence cardiovascular risk factors and arterial health in young adults: the Cardiovascular Risk in Young Finns Study. *Arterioscler Thromb Vasc Biol* 2011; **31**:2975–81.
14. Sandboge S, Osmond C, Kajantie E, Eriksson JG. Early growth and changes in blood pressure during adult life. *J Dev Orig Health Dis* 2015 Dec 10:1–8. [Epub ahead of print]
15. Soininen P, Kangas AJ, Würtz P, Suna T, Ala-Korpela M. Quantitative Serum Nuclear Magnetic Resonance Metabolomics in Cardiovascular Epidemiology and Genetics. *Circ Cardiovasc Genet* 2015; **8**:192–206.
16. Kettunen J, Demirkan A, Würtz P, et al. Genome-wide study for circulating metabolites identifies 62 loci and reveals novel systemic effects of LPA. *Nat Commun* 2016;**7**:11122.

**Table S1. Mean (SD) metabolic concentrations, and metabolic associations with birth weight in absolute concentration units.**

| **Metabolic measure** | **Mean (SD)** | **Association with birth weight**  **Beta [95%CI] per 1-kg lower birth weight; P-value** | **Association with BMI**  **Beta [95%CI] per kg/m^2^**  **P-value** | **n** |
| --- | --- | --- | --- | --- |
| **Lipoprotein subclass total lipids** | | | |  |
| Extremely large VLDL [mmol/L]* | 0.0155  (0.0235) | 0.120 [0.042– 0.198]  P=0.0004 | 0.120 [0.111– 0.129]  P=1×10^-158^ | 18249 |
| Very large VLDL [mmol/L]* | 0.0471  (0.0608) | 0.149 [0.0628– 0.235]  P=0.0004 | 0.132 [0.122– 0.142]  P=3×10^-150^ | 18247 |
| Large VLDL [mmol/L]* | 0.199  (0.219) | 0.129 [0.0648– 0.193]  P=1×10^-5^ | 0.0951 [0.0878– 0.102]  P=2×10^-143^ | 18250 |
| Medium VLDL [mmol/L]* | 0.459  (0.319) | 0.0546 [0.036– 0.0731]  P=2×10^-9^ | 0.0449 [0.0428– 0.0469]  P<1×10^-300^ | 18254 |
| Small VLDL [mmol/L] | 0.573  (0.238) | 0.0167 [0.0107– 0.0226]  P=7×10^-9^ | 0.0144 [0.0137– 0.015]  P<1×10^-300^ | 18254 |
| Very small VLDL [mmol/L] | 0.474  (0.146) | 0.00725 [0.00342– 0.0111] P=8×10^-5^ | 0.00665 [0.00621– 0.00709]  P=2×10^-196^ | 18252 |
| IDL [mmol/L] | 1.08  (0.33) | 0.0102 [0.00194– 0.0185]  P=0.009 | 0.0106 [0.00968– 0.0116]  P=3×10^-106^ | 18254 |
| Large LDL [mmol/L] | 1.32  (0.453) | 0.0149 [0.00439– 0.0254]  P=0.003 | 0.014 [0.0128– 0.0152]  P=1×10^-114^ | 18254 |
| Medium LDL [mmol/L] | 0.764  (0.289) | 0.0108 [0.00429– 0.0173]  P=0.0006 | 0.00958 [0.00884– 0.0103]  P=2×10^-140^ | 18254 |
| Small LDL [mmol/L] | 0.477  (0.183) | 0.00787 [0.00366– 0.0121]  P=8×10^-5^ | 0.00686 [0.00638– 0.00734]  P=2×10^-173^ | 18254 |
| Very large HDL [mmol/L] | 0.427  (0.217) | -0.00905 [-0.015– -0.00315]  P=0.002 | -0.0102 [-0.0109– -0.00952]  P=6×10^-196^ | 18253 |
| Large HDL [mmol/L] | 0.812  (0.366) | -0.0177 [-0.0266– -0.00875]  P=6×10^-5^ | -0.02 [-0.021– -0.019]  P<1×10^-300^ | 18253 |
| Medium HDL [mmol/L] | 0.921  (0.249) | 0.00355 [-0.00222– 0.00932]  P=0.12 | -0.0019 [-0.0026– -0.0013]  P=1×10^-8^ | 18254 |
| Small HDL [mmol/L] | 1.15  (0.181) | 0.00956 [0.00514– 0.014]  P=1×10^-5^ | 0.00357 [0.00306– 0.00408]  P=3×10^-43^ | 18254 |
| **Lipoprotein particle size** | | | | |
| VLDL particle size [nm] | 36.3  (1.35) | 0.115 [0.0739– 0.156]  P=2×10^-8^ | 0.0874 [0.0828– 0.0919]  P<1×10^-300^ | 18254 |
| LDL particle size [nm] | 23.6  (0.182) | -0.00922 [-0.0144– -0.00408]  P=0.0002 | -0.00421 [-0.0048– -0.0036]  P=3×10^-45^ | 18249 |
| HDL particle size [nm] | 9.93  (0.24) | -0.016 [-0.0226– -0.00942]  P=9×10^-7^ | -0.0161 [-0.0168– -0.0154]  P<1×10^-300^ | 18253 |
| **Apolipoproteins** | | | | |
| Apolipoprotein B [g/L] | 0.865  (0.245) | 0.0128 [0.00675– 0.0188]  P=2×10^-5^ | 0.0129 [0.0123– 0.0136]  P<1×10^-300^ | 17423 |
| Apolipoprotein A-I [g/L] | 1.6  (0.258) | 0.00222 [-0.00393– 0.00838]  P=0.31 | -0.00496 [-0.0056– -0.0042]  P=2×10^-43^ | 17423 |
| Apolipoprotein B/  apolipoprotein A-I | 0.545  (0.141) | 0.00721 [0.00325– 0.0112]  P=0.0004 | 0.0106 [0.0101– 0.011]  P<1×10^-300^ | 17423 |
| **Cholesterol (C)** | | | | |
| Total C [mmol/L] | 4.66  (1.21) | 0.038 [0.00984– 0.0661]  P=0.006 | 0.0239 [0.0207– 0.0272]  P=1×10^-47^ | 18254 |
| Non-HDL C [mmol/L] | 3.1  (1.07) | 0.0461 [0.0206– 0.0716]  P=0.0003 | 0.0424 [0.0395– 0.0453]  P=3×10^-180^ | 18254 |
| Remnant C [mmol/L] | 1.36  (0.431) | 0.0222 [0.011– 0.0333]  P=7×10^-5^ | 0.021 [0.0197– 0.0222]  P=3×10^-234^ | 18254 |
| VLDL C [mmol/L] | 0.669  (0.262) | 0.0164 [0.0094– 0.0234]  P=3×10^-6^ | 0.0152 [0.0144– 0.016]  P<1×10^-300^ | 18254 |
| IDL C [mmol/L] | 0.698  (0.212) | 0.0057 [0.000356– 0.0111]  P=0.03 | 0.00589 [0.00527– 0.0065]  P=2×10^-78^ | 18254 |
| LDL C [mmol/L] | 1.73  (0.675) | 0.023 [0.00762– 0.0384]  P=0.002 | 0.0209 [0.0191– 0.0227]  P=1×10^-119^ | 18254 |
| HDL C [mmol/L] | 1.56  (0.385) | -0.00803 [-0.0178– 0.00171]  P=0.10 | -0.0172 [-0.0183– -0.0161]  P=1×10^-203^ | 18253 |
| Esterified C [mmol/L] | 3.37  (0.922) | 0.0278 [0.00719– 0.0484]  P=0.005 | 0.018 [0.0156– 0.0203]  P=6×10^-50^ | 18071 |
| Free C [mmol/L] | 1.29  (0.312) | 0.00783 [-0.000334– 0.016]  P=0.06 | 0.00614 [0.0052– 0.00708]  P=3×10^-37^ | 18065 |
| Esterification [%] | 72.2  (2.33) | 0.0935 [0.0373– 0.15]  P=0.002 | 0.0184 [0.0119– 0.0248]  P=3×10^-8^ | 18071 |
| **Triglycerides (TG)** | | | | |
| Total triglycerides [mmol/L]* | 1.09  (0.557) | 0.0432 [0.0301– 0.0563]  P=4×10^-11^ | 0.0334 [0.032– 0.0349]  P<1×10^-300^ | 18252 |
| VLDL TG [mmol/L]* | 0.68  (0.453) | 0.0601 [0.0418– 0.0784]  P=1×10^-11^ | 0.0446 [0.0426– 0.0467]  P<1×10^-300^ | 18248 |
| IDL TG [mmol/L] | 0.104  (0.0399) | 0.0019 [0.000895– 0.00291]  P=4×10^-5^ | 0.0016 [0.00149– 0.00172]  P=2×10^-163^ | 18254 |
| LDL TG [mmol/L] | 0.201  (0.0912) | 0.00482 [0.00267– 0.00697]  P=8×10^-7^ | 0.00297 [0.00273– 0.00321]  P=6×10^-127^ | 18249 |
| HDL TG [mmol/L] | 0.0997  (0.0511) | 0.000845 [-0.00033– 0.0020]  P=0.17 | 0.00129 [0.00116– 0.00142]  P=1×10^-80^ | 18253 |
| **Phospholipids (PL)** | | | | |
| Total phospholipids [mmol/L] | 2.96  (0.611) | 0.0151 [0.000414– 0.0298]  P=0.04 | 0.00632 [0.00463– 0.00801]  P=2×10^-13^ | 18254 |
| VLDL PL [mmol/L] | 0.405  (0.185) | 0.0127 [0.00786– 0.0175]  P=4×10^-8^ | 0.0121 [0.0116– 0.0127]  P<1×10^-300^ | 18254 |
| IDL PL [mmol/L] | 0.284  (0.0874) | 0.00218 [6.1e-06– 0.00436]  P=0.02 | 0.00255 [0.0023– 0.0028]  P=3×10^-89^ | 18254 |
| LDL PL [mmol/L] | 0.624  (0.196) | 0.00539 [0.000773– 0.01]  P=0.01 | 0.00622 [0.0057– 0.00675]  P=2×10^-119^ | 18252 |
| HDL PL [mmol/L] | 1.64  (0.381) | -0.00676 [-0.0164– 0.00291]  P=0.20 | -0.0144 [-0.0155– -0.0133]  P=1×10^-144^ | 18254 |
| Phosphoglycerides [mmol/L] | 1.78  (0.429) | 0.0213 [0.00938– 0.0333]  P=0.0003 | 0.00462 [0.00324– 0.006]  P=5×10^-11^ | 18083 |
| Cholines [mmol/L] | 1.99  (0.451) | 0.0235 [0.0119– 0.0351]  P=0.0001 | 0.00237 [0.00101– 0.00374]  P=0.0007 | 18055 |
| Sphingomyelin [mmol/L] | 0.379  (0.109) | 0.0024 [-0.000185– 0.00498]  P=0.05 | 0.00169 [0.0014– 0.00198]  P=3.8×10^-31^ | 15185 |
| **Fatty acids** | | | | |
| Total fatty acids [mmol/L] | 11  (2.84) | 0.197 [0.122– 0.272]  P=5×10^-8^ | 0.121 [0.112– 0.129]  P=1×10^-168^ | 18092 |
| Saturated fatty acids [mmol/L] | 4.01  (1.11) | 0.082 [0.0521– 0.112]  P=1×10^-8^ | 0.0464 [0.0431– 0.0498]  P=1×10^-159^ | 18090 |
| MUFA [mmol/L] | 2.97  (1.04) | 0.062 [0.0356– 0.0884]  P=5×10^-7^ | 0.0514 [0.0484– 0.0544]  P=6×10^-243^ | 18103 |
| PUFA [mmol/L] | 4.04  (0.949) | 0.0492 [0.0254– 0.0731]  P=1×10^-5^ | 0.0225 [0.0197– 0.0252]  P=2×10^-58^ | 18092 |
| Omega-6 fatty acids [mmol/L] | 3.64  (0.848) | 0.0422 [0.0208– 0.0637]  P=4×10^-5^ | 0.019 [0.0165– 0.0215]  P=1×10^-51^ | 18102 |
| Linoleic acid [mmol/L] | 2.98  (0.724) | 0.0388 [0.0208– 0.0568]  P=1×10^-5^ | 0.0103 [0.0082– 0.0124]  P=3×10^-22^ | 18093 |
| Omega-3 fatty acids [mmol/L] | 0.401  (0.152) | 0.00665 [0.00302– 0.0103]  P=0.0001 | 0.00354 [0.00312– 0.00396]  P=2×10^-61^ | 18111 |
| Docosahexaenoic acid [mmol/L] | 0.159  (0.0703) | 0.00185 [0.000217– 0.00349]  P=0.01 | 0.000886 [0.00069– 0.0011]  P=1×10^-19^ | 18057 |
| **Fatty acid ratios, relative to total fatty acids** | | | | |
| Saturated fatty acids [%] | 36.4  (3.89) | 0.11 [0.0456– 0.174]  P=0.006 | 0.0178 [0.0102– 0.0255]  P=5×10^-6^ | 18084 |
| MUFA [%] | 26.6  (3.77) | 0.104 [0.0103– 0.198]  P=0.009 | 0.166 [0.155– 0.177]  P=3×10^-198^ | 18085 |
| PUFA [%] | 37  (3.53) | -0.195 [-0.292– -0.0979]  P=4×10^-5^ | -0.179 [-0.19– -0.168]  P=2×10^-214^ | 18093 |
| Omega-6 fatty acids [%] | 33.4  (3.44) | -0.187 [-0.279– -0.095]  P=2×10^-5^ | -0.17 [-0.181– -0.16]  P=1×10^-214^ | 18104 |
| Linoleic acid [%] | 27.3  (3.84) | -0.117 [-0.215– -0.0193]  P=0.007 | -0.19 [-0.201– -0.179]  P=1×10^-242^ | 18073 |
| Omega-3 fatty acids [%] | 3.63  (0.933) | -0.00389 [-0.029– 0.0212]  P=0.83 | -0.00587 [-0.0088– -0.0029]  P=9×10^-5^ | 18104 |
| Docosahexaenoic acid [%] | 1.43  (0.482) | -0.00745 [-0.0194– 0.00449]  P=0.29 | -0.00541 [-0.0068– -0.0040]  P=8×10^-14^ | 18039 |
| Unsaturation degree | 1.21  (0.0875) | -0.0031 [-0.0053– -0.000891]  P=0.006 | -0.00216 [-0.0024– -0.0019]  P=3×10^-60^ | 18102 |
| **Amino acids** | | | | |
| Alanine [µmol/L] | 394  (100) | 5.26 [3.14– 7.38]  P=1×10^-6^ | 2.33 [2.09– 2.57]  P=1×10^-80^ | 18225 |
| Glutamine [µmol/L] | 548  (87.9) | -3.45 [-5.9– -0.993]  P=0.003 | -1.69 [-1.97– -1.4]  P=2×10^-31^ | 17691 |
| Glycine [µmol/L] | 306  (67.5) | -3.48 [-5.57– -1.4]  P=0.002 | -0.914 [-1.16– -0.67]  P=2×10^-13^ | 15270 |
| Histidine [µmol/L] | 69.2  (12.7) | 0.072 [-0.294– 0.438]  P=0.88 | 0.126 [0.0841– 0.168]  P=4×10^-9^ | 18210 |
| Isoleucine [µmol/L] | 53.5  (16.2) | 1.18 [0.742– 1.62]  P=7.1×10^-8^ | 1.05 [0.998– 1.09]  P<1×10^-300^ | 18217 |
| Leucine [µmol/L] | 82.5  (21.4) | 1.05 [0.593– 1.52]  P=7×10^-7^ | 0.975 [0.924– 1.03]  P=2×10^-303^ | 18210 |
| Valine [µmol/L] | 202  (49.5) | 2.37 [1.2– 3.55]  P=3×10^-5^ | 2.31 [2.18– 2.44]  P=3×10^-263^ | 18220 |
| Phenylalanine [µmol/L] | 72.1  (20.2) | 0.435 [0.113– 0.757]  P=0.0006 | 0.7 [0.665– 0.736]  P<1×10^-300^ | 18222 |
| Tyrosine [µmol/L] | 54  (12.6) | 0.466 [0.092– 0.839]  P=0.01 | 0.764 [0.723– 0.805]  P=3×10^-287^ | 18226 |
| **Glycolysis and gluconeogenesis** | | | | |
| Glucose [mmol/L]* | 4.66  (0.881) | 0.00367 [-0.000407– 0.00775]  P=0.07 | 0.00362 [0.00315– 0.00408]  P=2×10^-52^ | 18214 |
| Lactate [mmol/L] | 1.43  (0.462) | 0.0225 [0.00971– 0.0352]  P=0.0005 | 0.00741 [0.00594– 0.00888]  P=5×10^-23^ | 18215 |
| Pyruvate [µmol/L] | 84.3  (27.1) | 2.12 [1.29– 2.95]  P=5×10^-6^ | 0.701 [0.605– 0.797]  P=3×10^-46^ | 15338 |
| Glycerol [µmol/L] | 81.5  (35.3) | 1.93 [0.828– 3.03]  P=0.0002 | 1.53 [1.4– 1.66]  P=2×10^-123^ | 15164 |
| **Ketone bodies** | | | | |
| Acetoacetate [µmol/L]* | 49.4  (41.8) | 0.0177 [-0.00279– 0.0381]  P=0.10 | -0.00657 [-0.0089– -0.0042]  P=3×10^-8^ | 18169 |
| Beta-hydroxybutyrate [µmol/L] | 94.1  (41.5) | 0.129 [-1.16– 1.42]  P=0.77 | -0.355 [-0.503– -0.206]  P=3×10^-6^ | 15334 |
| **Miscellaneous** | | | | |
| Citrate [µmol/L] | 103  (22) | 0.157 [-0.509– 0.823]  P=0.75 | -0.383 [-0.458– -0.307]  P=3×10^-23^ | 18191 |
| Acetate [µmol/L] | 44.4  (11.5) | 0.442 [0.0941– 0.789]  P=0.02 | -0.226 [-0.267– -0.186]  P=5×10^-28^ | 18178 |
| Creatinine [µmol/L] | 61.4  (12.7) | -0.194 [-0.519– 0.131]  P=0.33 | 0.0837 [0.0462– 0.121]  P=1×10^-5^ | 18203 |
| Albumin [cu] | 101  (13.9) | 0.219 [-0.0485– 0.487]  P=0.52 | -0.0776 [-0.108– -0.0472]  P=5×10^-7^ | 18254 |
| **Inflammatory markers** | | | | |
| Glycoprotein acetyls [mmol/L] | 1.37  (0.252) | 0.019 [0.0125– 0.0255]  P=4×10^-9^ | 0.0148 [0.0141– 0.0156]  P<1×10^-300^ | 18226 |
| C-reactive protein [mg/L]* | 1.44  (3.04) | 0.0518 [0.00349– 0.1]  P=0.04 | 0.124 [0.119– 0.129]  P<1×10^-300^ | 15906 |
| **Liver function markers** | | | | |
| Alanine aminotransferase [cu]* | 10  (1.0) | 0.00282 [0.000213– 0.00542]  P=0.12 | 0.00431 [0.00401– 0.0046]  P=8×10^-179^ | 11014 |
| Gamma-glutamine aminotransferase [cu]* | 10  (1.0) | 0.00548 [0.00282– 0.00814]  P=4.6×10^-5^ | 0.00475 [0.00445– 0.00504]  P=7×10^-219^ | 12631 |
| Aspartate aminotransferase [cu]* | 10.3  (2.44) | 0.00461 [0.00153– 0.00768] P=0.003 | 0.00184 [0.00149– 0.0022] P=3×10^-24^ | 7355 |
| Bilirubin [cu]* | 10  (1.0) | -0.00321 [-0.0070– 0.00055]  P=0.10 | -0.00281 [-0.0033– -0.0023]  P=9×10^-32^ | 10229 |
| **Hormone related** | | | | |
| Testosterone (Men)  [nmol/L] | 21  (6.6) | -0.28 [-0.643– 0.0843]  P=0.13 | -0.526 [-0.572– -0.48]  P=5×10^-112^ | 5643 |
| Testosterone (Women) [nmol/L] | 1.85  (0.835) | -0.00196 [-0.0571– 0.0531]  P=0.98 | 0.0265 [0.0203– 0.0328]  P=1×10^-16^ | 4204 |
| Sex-hormone binding globulin (Men) [nmol/L] | 33  (14) | -1.3 [-2.06– -0.546]  P=0.0007 | -1.52 [-1.61– -1.43]  P=3×10-233 | 5649 |
| Sex-hormone binding globulin (Women) [nmol/L] | 62  (35) | -0.526 [-2.72– 1.67]  P=0.63 | -2.22 [-2.44– -1.99]  P=3×10^-84^ | 4526 |
| Insulin [IU/L]* | 9.1  (5.1) | 0.0426 [0.0282– 0.0569]  P=5×10^-9^ | 0.0499 [0.0484– 0.0515]  P<1×10^-300^ | 16494 |

Mean and standard (SD) concentrations of the metabolic measures are from combined analyses pooled across the seven cohorts. The metabolic associations with birth weight and BMI in absolute concentration units were analysed separately in each cohort, with adjustment for age, sex, and gestational age, and subsequently meta-analysed. The absolute concentrations units correspond to the associations in Figures 1–3 multiplied by the SD of each individual metabolic measure. n indicates the number of individuals included in the analyses of each measure.

*: Metabolic measures log-transformed for the linear regression analyses in Figures 1–3 and the association magnitudes reported here; mean and SD are reported for non-transformed levels.

Seventy-seven metabolic measures were quantified using a high‑throughput serum NMR metabolomics platform^15^ and 10 metabolic measures were quantified by routine clinical chemistry. The 14 lipoprotein subclasses quantified were defined according to their size as follows: extremely large VLDL with particle diameters from 75 nm upwards and a possible contribution of chylomicrons, five VLDL subclasses (average particle diameters of 64.0 nm, 53.6 nm, 44.5 nm, 36.8 nm, and 31.3 nm), IDL (28.6 nm), three LDL subclasses (25.5 nm, 23.0 nm, and 18.7 nm), and four HDL subclasses (14.3 nm, 12.1 nm, 10.9 nm, and 8.7 nm). The mean size for VLDL, LDL and HDL particles was calculated by weighting the corresponding subclass diameters with their particle concentrations.

Representative coefficients of variations (CVs) over thousands of samples for the NMR-based metabolic measures are: Total cholesterol 2.1%, LDL-C 2.3%, HDL-C 2.3%, total triglycerides 1.2%, omega-6 fatty acids 4.5%, omega-6 fatty acid ratio (relative to total fatty acids) 2.2%, apolipoprotein A-I 1.6%, apolipoprotein B 2.2%, glucose 2.6%, lactate 3.2%, alanine 2.3%, glutamine 2.7%, isoleucine 3.4%, phenylalanine 3.9%, acetoacetate 3.6%, GlycA 1.9%. CVs for other metabolic measures are typically below 5%.^16^

Abbreviations: cu, standardized concentration unit; VLDL, very-low-density lipoprotein; IDL, intermediate-density lipoprotein; LDL, low-density lipoprotein; HDL, high-density lipoprotein; C, cholesterol; TG, triglycerides; PL, phospholipids; PUFA, polyunsaturated fatty acids; MUFA, monounsaturated fatty acids.

**Table S2. Mean (SD) concentrations of metabolites in each cohort.**

| **Metabolic measure**  **[Mean (SD) concentration]** | **NFBC 1986** | **ALSPAC Children** | **Finn-Twin**  **FT12** | **Finn-Twin**  **FT16** | **NFBC 1966** | **Young Finns Study** | **Helsinki Birth Cohort Study** |
| --- | --- | --- | --- | --- | --- | --- | --- |
| **Lipoprotein subclass total lipids** | | | | | | | |
| Extremely large VLDL [mmol/L]* | 0.0129 (0.0199) | 0.0176 (0.017) | 0.00913 (0.0148) | 0.0249 (0.0364) | 0.0173 (0.0267) | 0.017 (0.0286) | 0.00994 (0.0197) |
| Very large VLDL [mmol/L]* | 0.0469 (0.052) | 0.0358 (0.0412) | 0.0318 (0.0439) | 0.0698 (0.0785) | 0.0519 (0.0689) | 0.0511 (0.0763) | 0.0467 (0.0598) |
| Large VLDL [mmol/L]* | 0.185 (0.178) | 0.142 (0.128) | 0.178 (0.174) | 0.299 (0.286) | 0.213 (0.247) | 0.252 (0.285) | 0.212 (0.233) |
| Medium VLDL [mmol/L]* | 0.411 (0.248) | 0.353 (0.176) | 0.44 (0.266) | 0.612 (0.38) | 0.494 (0.354) | 0.582 (0.426) | 0.516 (0.363) |
| Small VLDL [mmol/L] | 0.503 (0.182) | 0.491 (0.119) | 0.44 (0.197) | 0.588 (0.234) | 0.65 (0.269) | 0.626 (0.271) | 0.767 (0.269) |
| Very small VLDL [mmol/L] | 0.414 (0.114) | 0.444 (0.0845) | 0.421 (0.128) | 0.463 (0.128) | 0.519 (0.162) | 0.522 (0.146) | 0.613 (0.169) |
| IDL [mmol/L] | 0.959 (0.248) | 0.838 (0.197) | 1.07 (0.265) | 1.14 (0.271) | 1.22 (0.347) | 1.23 (0.298) | 1.42 (0.315) |
| Large LDL [mmol/L] | 1.16 (0.313) | 0.87 (0.246) | 1.30 (0.333) | 1.43 (0.356) | 1.54 (0.461) | 1.52 (0.385) | 1.76 (0.379) |
| Medium LDL [mmol/L] | 0.668 (0.195) | 0.463 (0.150) | 0.747 (0.209) | 0.845 (0.231) | 0.917 (0.292) | 0.900 (0.246) | 1.03 (0.233) |
| Small LDL [mmol/L] | 0.409 (0.13) | 0.308 (0.0936) | 0.464 (0.136) | 0.536 (0.15) | 0.574 (0.19) | 0.565 (0.163) | 0.620 (0.148) |
| Very large HDL [mmol/L] | 0.382 (0.187) | 0.508 (0.138) | 0.365 (0.263) | 0.333 (0.253) | 0.466 (0.224) | 0.362 (0.23) | 0.476 (0.298) |
| Large HDL [mmol/L] | 0.760 (0.291) | 0.715 (0.187) | 1.07 (0.452) | 0.971 (0.455) | 0.853 (0.421) | 0.812 (0.399) | 0.902 (0.45) |
| Medium HDL [mmol/L] | 0.846 (0.191) | 0.806 (0.12) | 1.22 (0.248) | 1.21 (0.248) | 0.932 (0.289) | 1.06 (0.213) | 0.935 (0.205) |
| Small HDL [mmol/L] | 1.07 (0.135) | 1.02 (0.105) | 1.27 (0.149) | 1.30 (0.150) | 1.20 (0.196) | 1.26 (0.146) | 1.24 (0.149) |
| **Lipoprotein particle size** | | | | | | | |
| VLDL particle size [nm] | 36.5 (1.3) | 36.2 (1.11) | 36.4 (1.38) | 37.2 (1.59) | 36.2 (1.38) | 36.5 (1.53) | 36 (1.31) |
| LDL particle size [nm] | 23.6 (0.185) | 23.7 (0.126) | 23.6 (0.168) | 23.5 (0.184) | 23.5 (0.174) | 23.6 (0.174) | 23.6 (0.162) |
| HDL particle size [nm] | 9.91 (0.222) | 9.98 (0.157) | 9.99 (0.264) | 9.93 (0.269) | 9.93 (0.255) | 9.89 (0.266) | 9.93 (0.318) |
| **Apolipoproteins** | | | | | | | |
| Apolipoprotein B [g/L] | 0.787 (0.178) | 0.661 (0.133) | 0.814 (0.189) | 0.920 (0.218) | 0.98 (0.250) | 0.964 (0.244) | 1.12 (0.219) |
| Apolipoprotein A-I [g/L] | 1.50 (0.223) | 1.41  (0.130) | 1.75 (0.259) | 1.78 (0.235) | 1.69 (0.252) | 1.69 (0.237) | 1.80 (0.232) |
| Apolipoprotein B/  apolipoprotein A-I | 0.529 (0.117) | 0.472 (0.0952) | 0.471 (0.12) | 0.524 (0.139) | 0.586 (0.152) | 0.578 (0.157) | 0.633 (0.149) |
| **Cholesterol (C)** | | | | | | | |
| Total C [mmol/L] | 4.21 (0.863) | 3.53 (0.673) | 4.68 (0.884) | 5.02 (0.914) | 5.33  (1.24) | 5.04 (0.958) | 5.95 (0.955) |
| Non-HDL C [mmol/L] | 2.76 (0.748) | 2.13 (0.603) | 2.86 (0.783) | 3.25 (0.857) | 3.66  (1.12) | 3.44 (0.938) | 4.21 (0.922) |
| Remnant C [mmol/L] | 1.23  (0.32) | 1.09 (0.271) | 1.18 (0.332) | 1.36 (0.374) | 1.56 (0.463) | 1.44 (0.407) | 1.84 (0.438) |
| VLDL C [mmol/L] | 0.605 (0.201) | 0.564 (0.163) | 0.510 (0.207) | 0.640 (0.255) | 0.767 (0.287) | 0.685 (0.279) | 0.913 (0.304) |
| IDL C [mmol/L] | 0.63 (0.157) | 0.528 (0.132) | 0.673 (0.162) | 0.720 (0.170) | 0.796 (0.222) | 0.756 (0.186) | 0.932 (0.194) |
| LDL C [mmol/L] | 1.52  (0.460) | 1.04 (0.355) | 1.68 (0.489) | 1.89 (0.534) | 2.10 (0.689) | 2.00 (0.572) | 2.37 (0.551) |
| HDL C [mmol/L] | 1.45 (0.295) | 1.40  (0.230) | 1.82 (0.435) | 1.76 (0.420) | 1.67 (0.438) | 1.60 (0.375) | 1.74 (0.445) |
| Esterified C [mmol/L] | 2.99 (0.631) | 2.48 (0.484) | 3.39 (0.632) | 3.61 (0.658) | 3.95 (0.930) | 3.64 (0.699) | 4.34 (0.687) |
| Free C [mmol/L] | 1.21 (0.239) | 1.04 (0.208) | 1.29 (0.252) | 1.41 (0.275) | 1.38 (0.343) | 1.40 (0.275) | 1.61 (0.292) |
| Esterification [%] | 71.1 (1.53) | 70.4 (2.03) | 72.5 (1.36) | 71.9 (1.48) | 74.2 (2.15) | 72.2 (1.62) | 73.0 (1.95) |
| **Triglycerides (TG)** | | | | | | | |
| Total triglycerides [mmol/L]* | 0.952 (0.433) | 0.91 (0.328) | 1.01 (0.461) | 1.33 (0.664) | 1.16 (0.623) | 1.34 (0.684) | 1.30 (0.605) |
| VLDL TG [mmol/L]* | 0.608 (0.363) | 0.59 (0.276) | 0.629 (0.389) | 0.897 (0.54) | 0.707 (0.506) | 0.843 (0.582) | 0.769 (0.52) |
| IDL TG [mmol/L] | 0.086 (0.0302) | 0.0889 (0.0243) | 0.101 (0.0366) | 0.108 (0.0359) | 0.111 (0.0416) | 0.134 (0.0387) | 0.145 (0.0436) |
| LDL TG [mmol/L] | 0.184 (0.0729) | 0.125 (0.0478) | 0.132 (0.0619) | 0.167 (0.0809) | 0.251 (0.0936) | 0.212 (0.0763) | 0.298 (0.0732) |
| HDL TG [mmol/L] | 0.0742 (0.0444) | 0.106 (0.0222) | 0.144 (0.0418) | 0.149 (0.0462) | 0.0928 (0.0519) | 0.15 (0.0425) | 0.0874 (0.0433) |
| **Phospholipids (PL)** | | | | | | | |
| Total phospholipids [mmol/L] | 2.67 (0.480) | 2.57 (0.347) | 3.34 (0.579) | 3.43 (0.545) | 3.14 (0.649) | 3.34 (0.496) | 3.33 (0.499) |
| VLDL PL [mmol/L] | 0.352 (0.142) | 0.328 (0.103) | 0.368 (0.15) | 0.469 (0.192) | 0.448 (0.208) | 0.485 (0.22) | 0.505 (0.199) |
| IDL PL [mmol/L] | 0.248 (0.0668) | 0.221 (0.0498) | 0.301 (0.0719) | 0.316 (0.0726) | 0.313 (0.0922) | 0.338 (0.0796) | 0.353 (0.0814) |
| LDL PL [mmol/L] | 0.534 (0.150) | 0.481 (0.100) | 0.707 (0.145) | 0.750 (0.157) | 0.688 (0.206) | 0.773 (0.168) | 0.746 (0.156) |
| HDL PL [mmol/L] | 1.54 (0.302) | 1.54 (0.230) | 1.96 (0.459) | 1.90 (0.435) | 1.69 (0.435) | 1.74 (0.371) | 1.73 (0.424) |
| Phosphoglycerides [mmol/L] | 1.83 (0.365) | 1.56 (0.303) | 1.70 (0.449) | 1.86 (0.451) | 1.82 (0.499) | 1.72  (0.39) | 2.07 (0.437) |
| Cholines [mmol/L] | 1.9 (0.314) | 1.68 (0.312) | 2.1 (0.473) | 2.23 (0.487) | 2.13 (0.525) | 2.03 (0.409) | 2.33 (0.448) |
| Sphingomyelin [mmol/L] | 0.468 (0.0842) | – | 0.285 (0.063) | 0.301 (0.0684) | 0.363 (0.0837) | 0.269 (0.058) | 0.321 (0.0728) |
| **Fatty acids** | | | | | | | |
| Total fatty acids [mmol/L] | 11 (2.36) | 9.03 (1.68) | 10.2 (2.43) | 11.9 (3.1) | 11.6 (3.16) | 11.2 (2.78) | 13.9 (2.6) |
| Saturated fatty acids [mmol/L] | 4.46  (0.99) | 3.45  (0.66) | 3.32  (0.95) | 4.12  (1.30) | 3.89  (1.18) | 3.76  (1.07) | 4.88  (1.00) |
| MUFA [mmol/L] | 2.68 (0.743) | 2.32 (0.591) | 3.00 (0.902) | 3.56  (1.18) | 3.30  (1.16) | 3.30  (1.16) | 3.74  (1.05) |
| PUFA [mmol/L] | 3.89 (0.734) | 3.27 (0.567) | 3.87 (0.772) | 4.21 (0.837) | 4.38 (1.03) | 4.12 (0.786) | 5.30 (0.872) |
| Omega-6 fatty acids [mmol/L] | 3.48 (0.654) | 2.97 (0.511) | 3.53 (0.705) | 3.83 (0.751) | 3.98 (0.941) | 3.70  (0.705) | 4.61 (0.784) |
| Linoleic acid [mmol/L] | 2.74 (0.541) | 2.39 (0.428) | 3.01 (0.585) | 3.28 (0.628) | 3.35  (0.79) | 3.13 (0.605) | 3.54 (0.626) |
| Omega-3 fatty acids [mmol/L] | 0.412 (0.109) | 0.298 (0.0783) | 0.336 (0.115) | 0.381 (0.141) | 0.401 (0.152) | 0.413 (0.139) | 0.696 (0.202) |
| Docosahexaenoic acid [mmol/L] | 0.148 (0.048) | 0.101 (0.0359) | 0.159 (0.065) | 0.167 (0.0734) | 0.176 (0.0728) | 0.171 (0.0662) | 0.266 (0.0888) |
| **Fatty acid ratios, relative to total fatty acids** | | | | | | | |
| Saturated fatty acids [%] | 40.4 (1.51) | 38.2 (2.12) | 32.3 (2.38) | 34.4 (4.08) | 33.5 (2.68) | 33.5 (2.47) | 35 (2.18) |
| MUFA [%] | 24.1 (2.32) | 25.5 (2.88) | 29.2 (3.26) | 29.6 (3.78) | 28.2 (3.66) | 29 (3.67) | 26.6 (3.49) |
| PUFA [%] | 35.5 (2.48) | 36.4 (2.57) | 38.4 (3.6) | 36 (4.11) | 38.3 (3.74) | 37.5 (4.24) | 38.4 (3.79) |
| Omega-6 fatty acids [%] | 31.8 (2.3) | 33.1 (2.43) | 35.1 (3.56) | 32.8 (4.09) | 34.8 (3.72) | 33.8 (4.07) | 33.4 (3.57) |
| Linoleic acid [%] | 25.1 (2.56) | 26.5 (2.43) | 30 (3.79) | 28.1 (4.07) | 29.5 (3.95) | 28.6 (3.93) | 25.7 (3.66) |
| Omega-3 fatty acids [%] | 3.75 (0.676) | 3.30 (0.615) | 3.31 (0.887) | 3.20 (0.781) | 3.49 (0.975) | 3.73 (0.996) | 5.05  (1.32) |
| Docosahexaenoic acid [%] | 1.34 (0.309) | 1.11 (0.285) | 1.55 (0.492) | 1.40 (0.494) | 1.55 (0.538) | 1.56 (0.532) | 1.93 (0.581) |
| Unsaturation degree | 1.15 (0.0566) | 1.18 (0.0641) | 1.26 (0.0735) | 1.21 (0.0829) | 1.26 (0.0819) | 1.26 (0.0859) | 1.22 (0.0869) |
| **Amino acids** | | | | | | | |
| Alanine [µmol/L] | 406 (64.9) | 231 (55.4) | 412 (61.4) | 494 (86.7) | 440 (81.6) | 413 (64.2) | 447 (56.5) |
| Glutamine [µmol/L] | 525 (71.7) | 540 (70) | 576 (88.6) | 556 (91.1) | 556 (103) | 593 (80.9) | 480 (76.9) |
| Glycine [µmol/L] | 285 (50.1) | – | 288 (56.5) | 294 (59.7) | 332 (73.3) | 301 (66.1) | 321 (83.9) |
| Histidine [µmol/L] | 65.1 (9.86) | 72.1 (15.1) | 74.6 (11.6) | 76.8 (15.2) | 70.6 (13.6) | 67.5 (10.5) | 71.4 (10.3) |
| Isoleucine [µmol/L] | 55 (13.1) | 42.6 (12.5) | 51 (12.7) | 65.8 (22.9) | 55.6 (17.6) | 56 (17.2) | 55.9 (15.2) |
| Leucine [µmol/L] | 84 (16.2) | 57.7 (11) | 80.3 (14.7) | 97.8 (28.6) | 91.9 (21.6) | 85.2 (19.1) | 83 (15.6) |
| Valine [µmol/L] | 206 (38.6) | 146 (30.1) | 199 (40.8) | 229 (54) | 223 (50) | 209 (42.9) | 198 (36.9) |
| Phenylalanine [µmol/L] | 70.6 (10.8) | 37.7 (5.99) | 78.8 (11.2) | 84.2 (15) | 85.5 (15.4) | 77.5 (12.5) | 84.1 (12.1) |
| Tyrosine [µmol/L] | 52 (11.4) | 53.2 (11.3) | 52.9 (12.3) | 67.7 (18.9) | 54.3 (13.2) | 53.2 (11.6) | 61.5 (11.2) |
| **Glycolysis and gluconeogenesis** | | | | | | | |
| Glucose [mmol/L]* | 4.57 (0.774) | 4.15 (0.518) | 4.65 (0.662) | 4.80  (1.16) | 4.85  (1.04) | 4.78 (0.828) | 5.23 (0.659) |
| Lactate [mmol/L] | 1.47 (0.344) | 0.984 (0.451) | 1.50 (0.347) | 1.59 (0.419) | 1.57 (0.477) | 1.49 (0.382) | 1.49 (0.466) |
| Pyruvate [µmol/L] | 74.6 (20.6) | – | 78.2 (24) | 104 (31.7) | 89.7 (27.2) | 82 (25.5) | 86 (26.5) |
| Glycerol [µmol/L] | 67.9 (27.8) | – | 75.2 (25) | 65.8 (32.1) | 88 (36) | 100 (37.9) | 92 (38.1) |
| **Ketone bodies** |  |  |  |  |  |  |  |
| Acetoacetate [µmol/L]* | 50.2 (43.5) | 37.1 (38.9) | 35.7 (31.3) | 31.8 (21.4) | 55.5 (42.3) | 55.7 (41.3) | 52.3 (40.3) |
| Beta-hydroxybutyrate [µmol/L] | 100 (35) | 65.7 (42.5) | 82.5 (38.7) | 67.7 (32.3) | 112 (36) | 78.4 (42.4) | 111 (33.3) |
|  |  |  |  |  |  |  |  |
| Citrate [µmol/L] | 103 (23.6) | 93.7 (22.4) | 106 (18.4) | 108 (19.4) | 106 (20) | 106 (19.6) | 94.7 (22) |
| Acetate [µmol/L] | 46.3 (9.87) | 42.1 (10.9) | 41.2 (9.71) | 37.2 (9.18) | 42.9 (11.6) | 46 (12.5) | 51.7 (15) |
| Creatinine [µmol/L] | 54.8 (9.77) | 64.4 (10.8) | 62.1 (11.5) | 61.5 (12.6) | 65.1 (13.7) | 63.9 (12.2) | 63.7 (14.2) |
| Albumin [cu] | 101 (12.4) | 89.3 (4.61) | 109 (7.55) | 109 (9.97) | 105 (17.6) | 105 (9.52) | 94.3 (7.1) |
| **Inflammatory markers** | | | | | | | |
| Glycoprotein acetyls [mmol/L] | 1.34 (0.215) | 1.21 (0.134) | 1.55 (0.21) | 1.46 (0.233) | 1.38 (0.266) | 1.55 (0.247) | 1.21 (0.285) |
| C-reactive protein [mg/L]* | 0.883 (2.52) | 1.37  (2.60) | – | – | 1.92  (3.69) | 1.72  (2.71) | – |
| **Liver function markers** | | | | | | | |
| Alanine aminotransferase [cu]* | 9.99 (0.957) | – | – | – | 9.99 (0.995) | 9.98 (0.824) | – |
| Gamma-glutamine aminotransferase [cu]* | 10.0 (0.993) | – | – | – | 10.0  (1.03) | 9.98 (0.931) | – |
| Aspartate aminotransferase [cu]* | 10.0  (1.05) | – | – | – | – | 9.98 (0.725) | – |
| Bilirubin [cu]* | 10.0 (0.983) | – | – | – | 10.0  (1.01) | – | – |
| **Hormone related** | | | | | | | |
| Testosterone (Men) [nmol/L] | 20.5  (6.59) | – | – | – | 21.7  (6.47) | 18.2  (6.62) | – |
| Testosterone (Women) [nmol/L] | 1.74 (0.807) | – | – | – | 2.05  (0.84) | 1.74 (0.916) | – |
| Sex-hormone binding globulin (Men) [nmol/L] | 31.7  (14.2) | – | – | – | 33.5  (13.3) | 31.7  (12.3) | – |
| Sex-hormone binding globulin (Women) [nmol/L] | 60.8  (37.5) | – | – | – | 63.1  (30.4) | 70.9  (51.2) | – |
| Insulin [IU/L]* | 10.6 (5.07) | 7.94 (5.02) | – | – | 8.32 (3.74) | 8.8 (6.59) | 9.13 (6.05) |

Mean and standard (SD) concentrations of the metabolic measures in each cohort. The metabolic measures were quantified from plasma in ALSPAC samples whereas all other cohorts were serum samples, and this the metabolite levels therefore deviate the most.

Abbreviations: cu, standardized concentration unit; VLDL, very-low-density lipoprotein; IDL, intermediate-density lipoprotein; LDL, low-density lipoprotein; HDL, high-density lipoprotein; C, cholesterol; TG, triglycerides; PL, phospholipids; PUFA, polyunsaturated fatty acids; MUFA, monounsaturated fatty acids.

**Figure S1. Birth weight distributions in each cohort.**


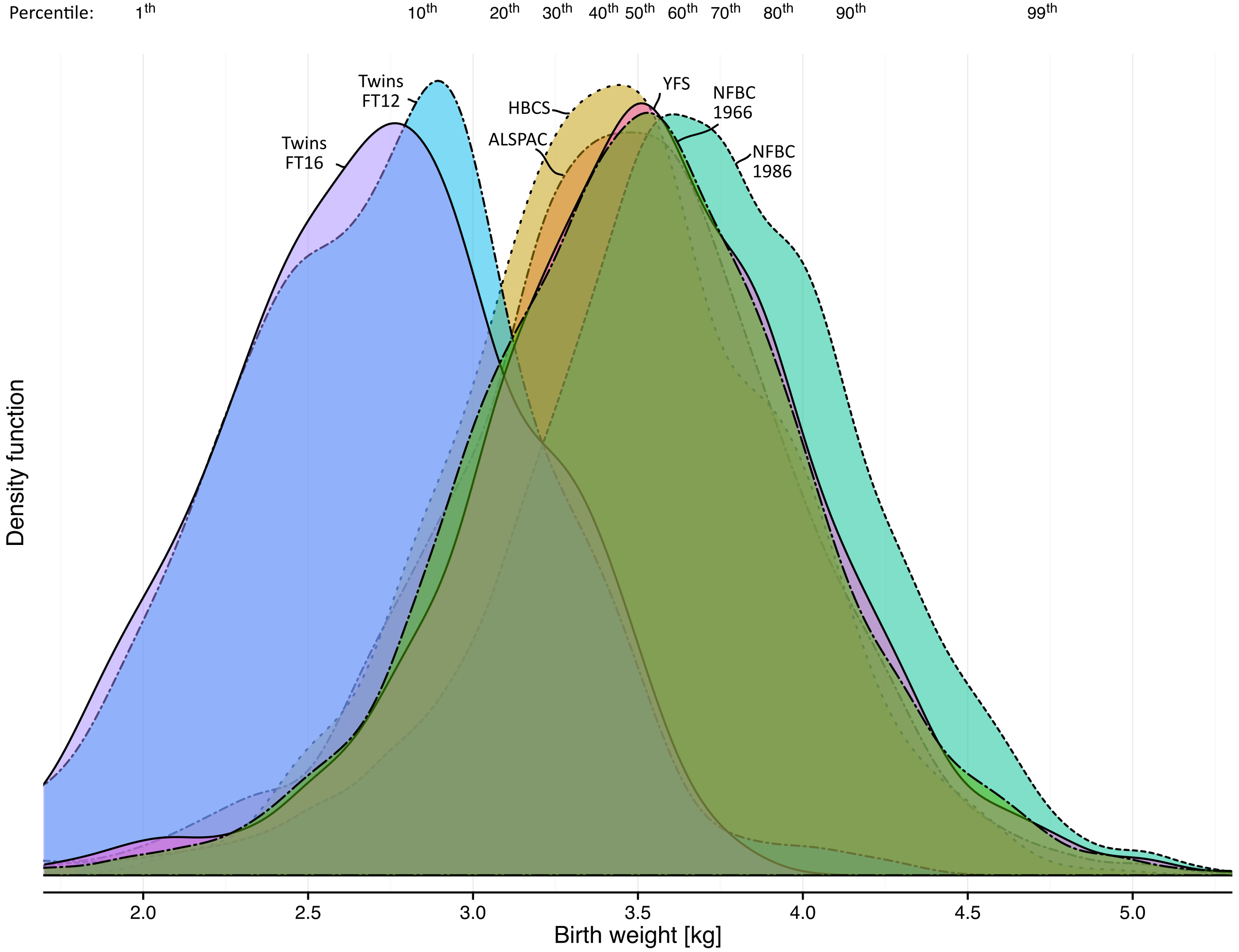
Density plots of birth weight for each cohort included in the study. Percentiles of birth weight for the five general population cohorts (i.e. non-twin cohorts; 93% of the study population) are indicated on the top.

**Figure S2. Adult body mass index as a function of birth weight in each cohort.**


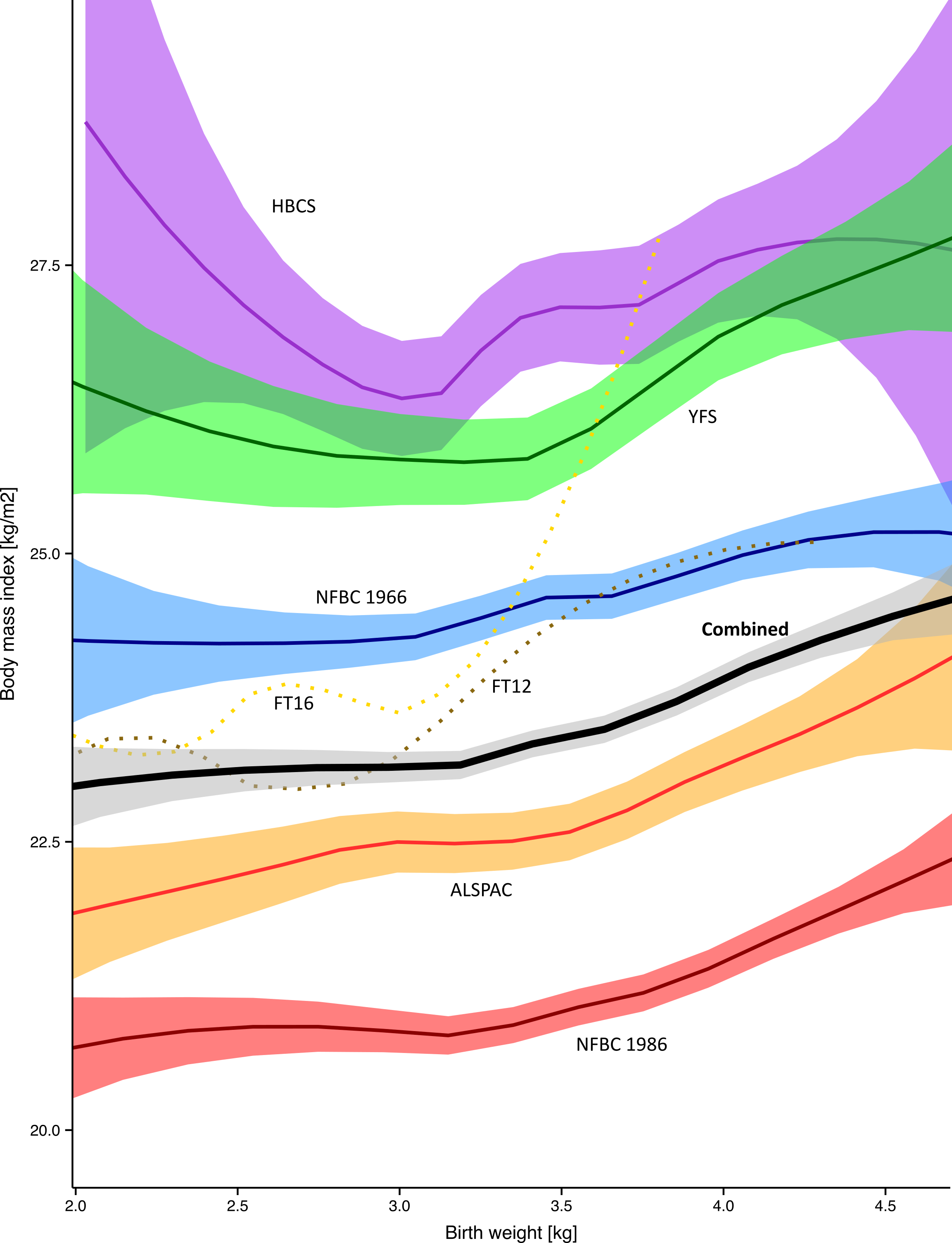


Shape of association between adult BMI as a function of birth weight. BMI was assessed at the same time as the blood sampling for metabolic profiling. The shaded curves denote the 95% confidence intervals of the local quadratic regression fits. These are not shown for the two twin cohorts due to the small size and different birth weight distributions. The thicker black line denotes the association shape when combining the 5 non-twin cohorts. Only two of the cohorts exhibit the phenonom of lower birth weight being associated with higher adult BMI.

**Figure S3. Metabolic associations with adulthood body mass index.**

Metabolic associations with adult body mass index (orange points) were adjusted for sex and age, and meta-analysed the whole study population (n=18 288). The association magnitudes are in units of SD metabolite concentration per 1-kg/m^2^ higher BMI. 95% confidence intervals are mostly within the orange bullet points. The metabolic associations with BMI have recently been demonstrated to be causal effects of higher adiposity.^5^ For comparison are shown the metabolic associations with birth weight for the same individuals (Figures 1–3). **Figure S4. Metabolic associations with adulthood height.**

Metabolic associations with adult height (green points) were adjusted for sex and age, and meta-analysed across the cohorts. The association magnitudes are in units of SD metabolite concentration per 9-cm lower height (corresponding to 1-SD lower adult height). Error bars indicate 95% confidence intervals. For comparison are shown the metabolic associations with birth weight for the same individuals (Figures 1–3).

**Figure S5A. Metabolic associations with birth weight in each cohort.**

Metabolic associations with birth weight as assessed in the 7 individual cohorts included in the study. Associations were adjusted for sex, age, and gestational age in each cohort and meta-analysed using fixed effect inverse-variance weighting (black diamonds). Error bars denote 95% confidence intervals. *I*^2^ indicates the percentage of variance in a meta-analysis that is attributable to study heterogeneity.

**Figure S5B. Metabolic associations with birth weight in each cohort.**

Metabolic associations with birth weight as assessed in the seven individual cohorts included in the study. Associations were adjusted for sex, age, and gestational age in each cohort and meta-analysed using fixed effect inverse-variance weighting (black diamonds). Error bars denote 95% confidence intervals. *I*^2^ indicates the percentage of variance in a meta-analysis that is attributable to study heterogeneity.**Figure S5C. Metabolic associations with birth weight in each cohort.**

Metabolic associations with birth weight as assessed in the 7 individual cohorts included in the study. Associations were adjusted for sex, age, and gestational age in each cohort and meta-analysed using fixed effect inverse-variance weighting (black diamonds). Error bars denote 95% confidence intervals. *I*^2^ indicates the percentage of variance in a meta-analysis that is attributable to study heterogeneity.

**Figure S6. Metabolic associations with birth weight for men and women.**

Metabolic associations with birth weight stratified by sex. The associations were adjusted for age and gestational age, and meta-analysed across the seven cohorts for men (n=8905) and women (n=9393). The standard deviation in birth weight was similar for men (580 g) and women (555 g).

**Figure S7. Metabolic associations with birth weight without adjustment for gestational age and gestational age as predictor of adulthood metabolic aberrations.**

Metabolic associations with birth weight compared with and without adjustment for gestational age. The associations were further adjusted for age and sex, and meta-analysed for the whole study population (n=18 288). The metabolic associations with gestational age as the primary exposure were adjusted for age and sex and meta-analysed for all cohorts except the YFS cohort (n=16 015), since this cohort did not have complete information on gestational age around term. Gestational age was analysed as a categorical variable of completed weeks of gestation in all the analyses.

**Figure S8. Metabolic associations with birth weight adjusted for adult BMI.**

Metabolic associations with birth weight compared with and without adjustment for adult BMI. BMI was assessed at the same time as the blood sampling for metabolic profiling. All associations were adjusted for age, sex and gestational age, and meta-analysed for the whole study population (n=18 288).

**Figure S9A. Curvilinear shapes of metabolic associations with birth weight.**

**
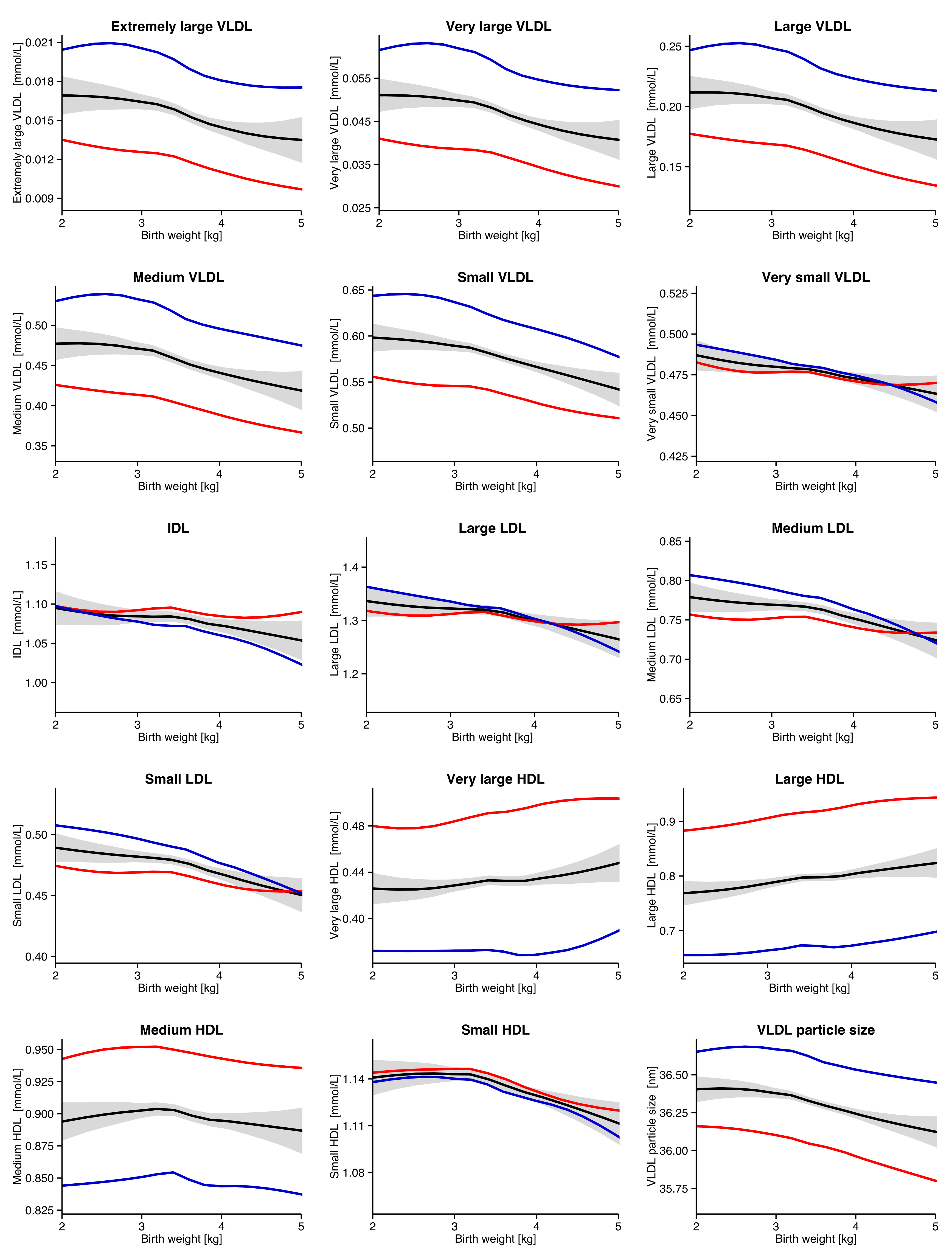
**

Continuous shape of the association between birth weight and 87 metabolic measures assessed in adulthood. Black curves denote the association shape for men and women combined (n=17 026), with the grey shaded area denoting the 95% confidence interval of the fit. Association shapes are depicted in blue for men (n=8336) and in red for women (n=8690). The association shapes were derived using local quadratic regression fitting evaluated at 25 points through the whole birth weight range. The metabolic measures were first adjusted for age and sex in each of the non-twin cohorts, and then pooled by combining the residuals and scaling back to absolute concentration units. Equivalent analyses were done stratified by sex. The 2 twin cohorts (n=1262) were omitted from these analyses due to their markedly lower distribution of birth weight (cf. Figure S1).

**Figure S9B. Curvilinear shapes of metabolic associations with birth weight.**


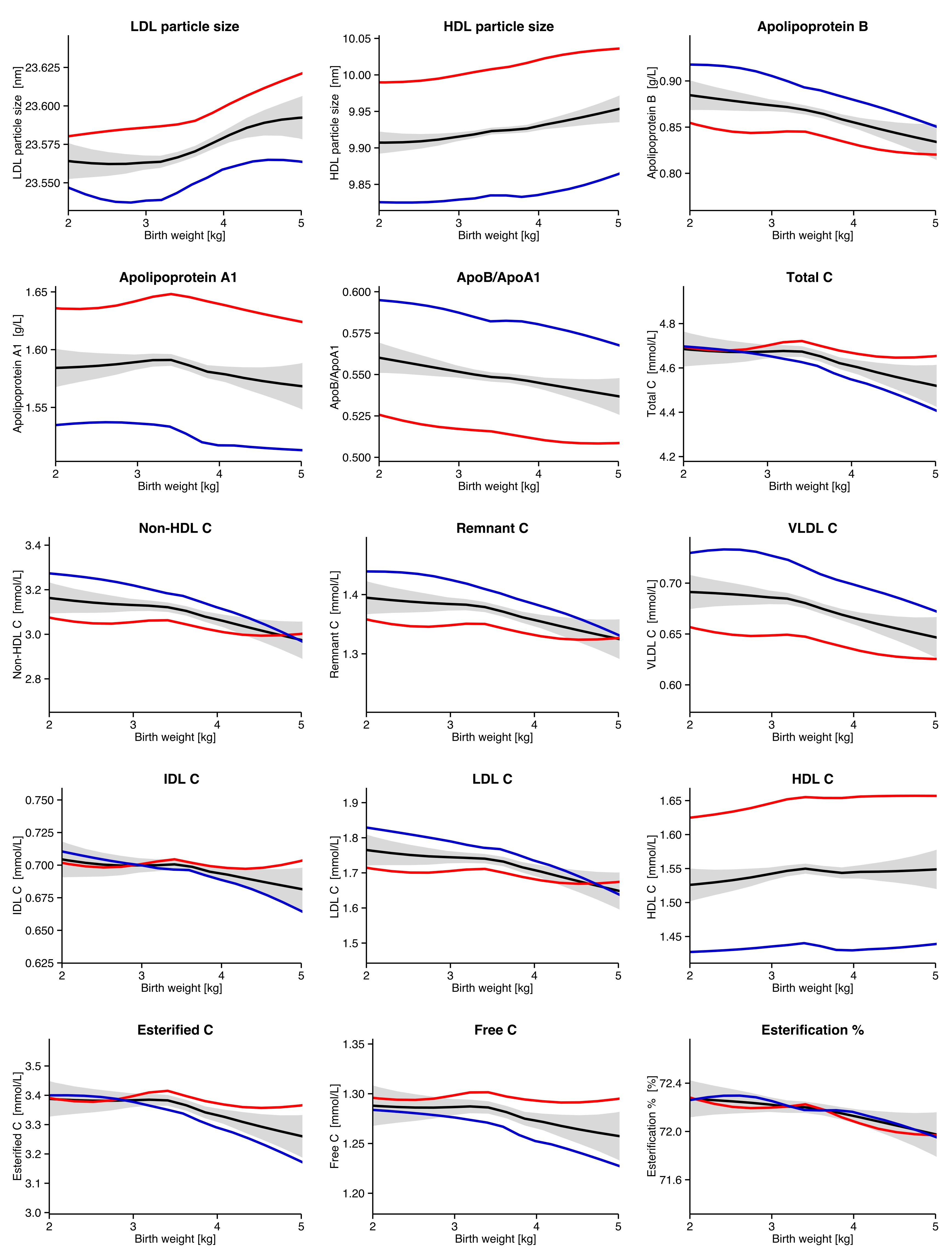


Continuous shape of the association between birth weight and 87 metabolic measures assessed in adulthood. Black curves denote the association shape for men and women combined (n=17 026), with the grey shaded area denoting the 95% confidence interval of the fit. Association shapes are depicted in blue for men (n=8336) and in red for women (n=8690). The association shapes were derived using local quadratic regression fitting evaluated at 25 points through the whole birth weight range. The metabolic measures were first adjusted for age and sex in each of the non-twin cohorts, and then pooled by combining the residuals and scaling back to absolute concentration units. Equivalent analyses were done stratified by sex. The 2 twin cohorts (n=1262) were omitted from these analyses due to their markedly lower distribution of birth weight (cf. Figure S1).

**Figure S9C. Curvilinear shapes of metabolic associations with birth weight.**


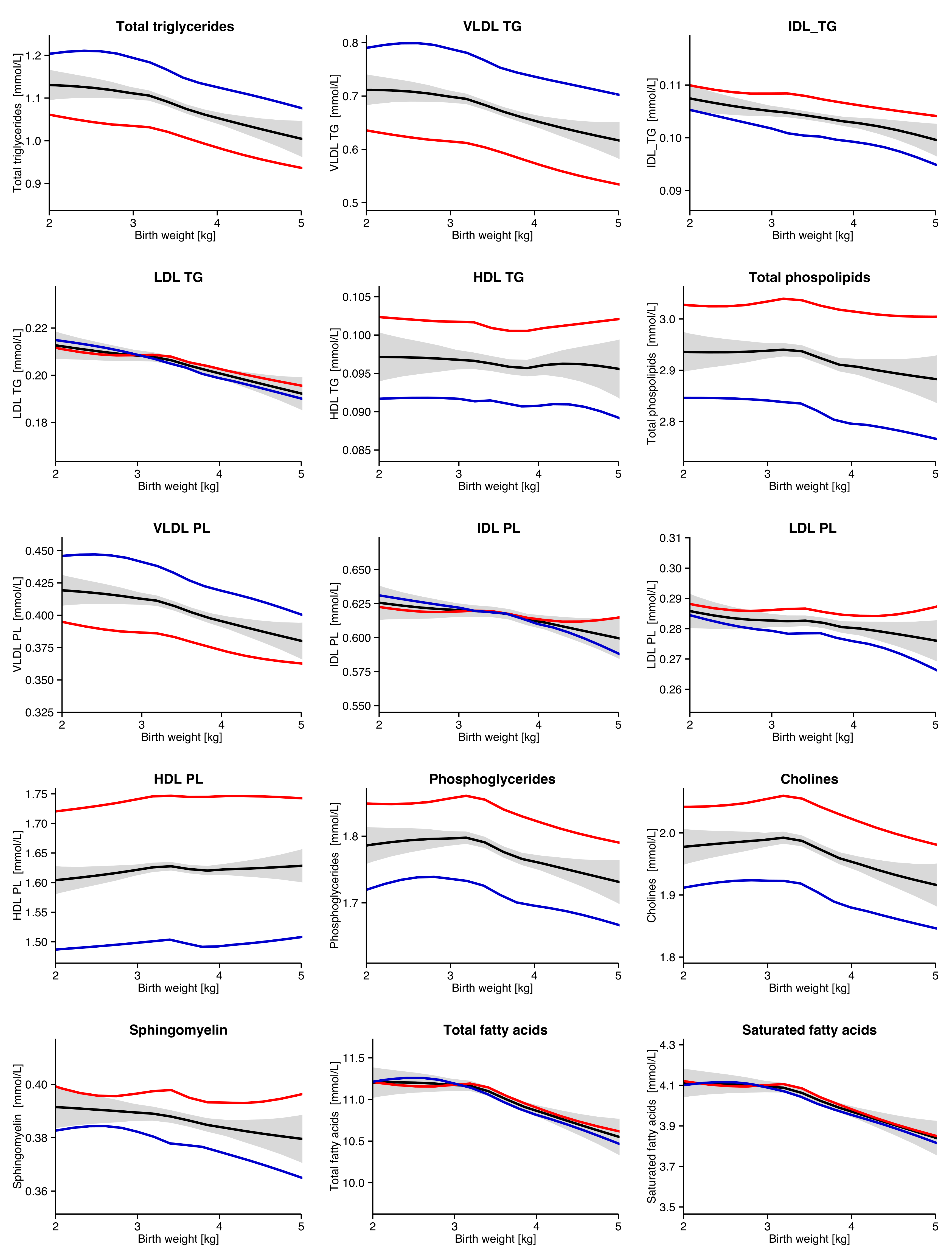


Continuous shape of the association between birth weight and 87 metabolic measures assessed in adulthood. Black curves denote the association shape for men and women combined (n=17 026), with the grey shaded area denoting the 95% confidence interval of the fit. Association shapes are depicted in blue for men (n=8336) and in red for women (n=8690). The association shapes were derived using local quadratic regression fitting evaluated at 25 points through the whole birth weight range. The metabolic measures were first adjusted for age and sex in each of the non-twin cohorts, and then pooled by combining the residuals and scaling back to absolute concentration units. Equivalent analyses were done stratified by sex. The 2 twin cohorts (n=1262) were omitted from these analyses due to their markedly lower distribution of birth weight (cf. Figure S1).

**Figure S9D. Curvilinear shapes of metabolic associations with birth weight.**


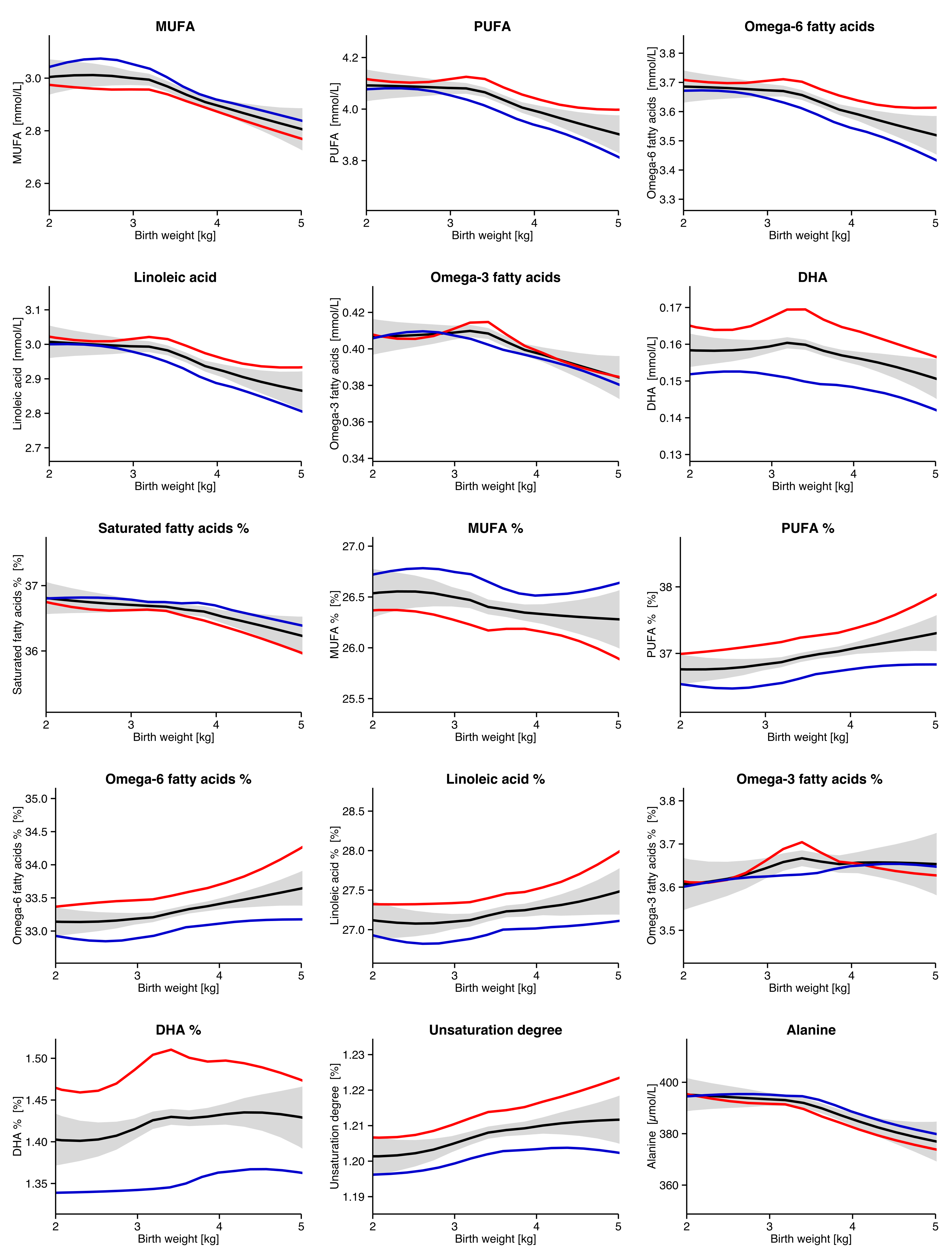


Continuous shape of the association between birth weight and 87 metabolic measures assessed in adulthood. Black curves denote the association shape for men and women combined (n=17 026), with the grey shaded area denoting the 95% confidence interval of the fit. Association shapes are depicted in blue for men (n=8336) and in red for women (n=8690). The association shapes were derived using local quadratic regression fitting evaluated at 25 points through the whole birth weight range. The metabolic measures were first adjusted for age and sex in each of the non-twin cohorts, and then pooled by combining the residuals and scaling back to absolute concentration units. Equivalent analyses were done stratified by sex. The 2 twin cohorts (n=1262) were omitted from these analyses due to their markedly lower distribution of birth weight (cf. Figure S1).

**Figure S9E. Curvilinear shapes of metabolic associations with birth weight.**


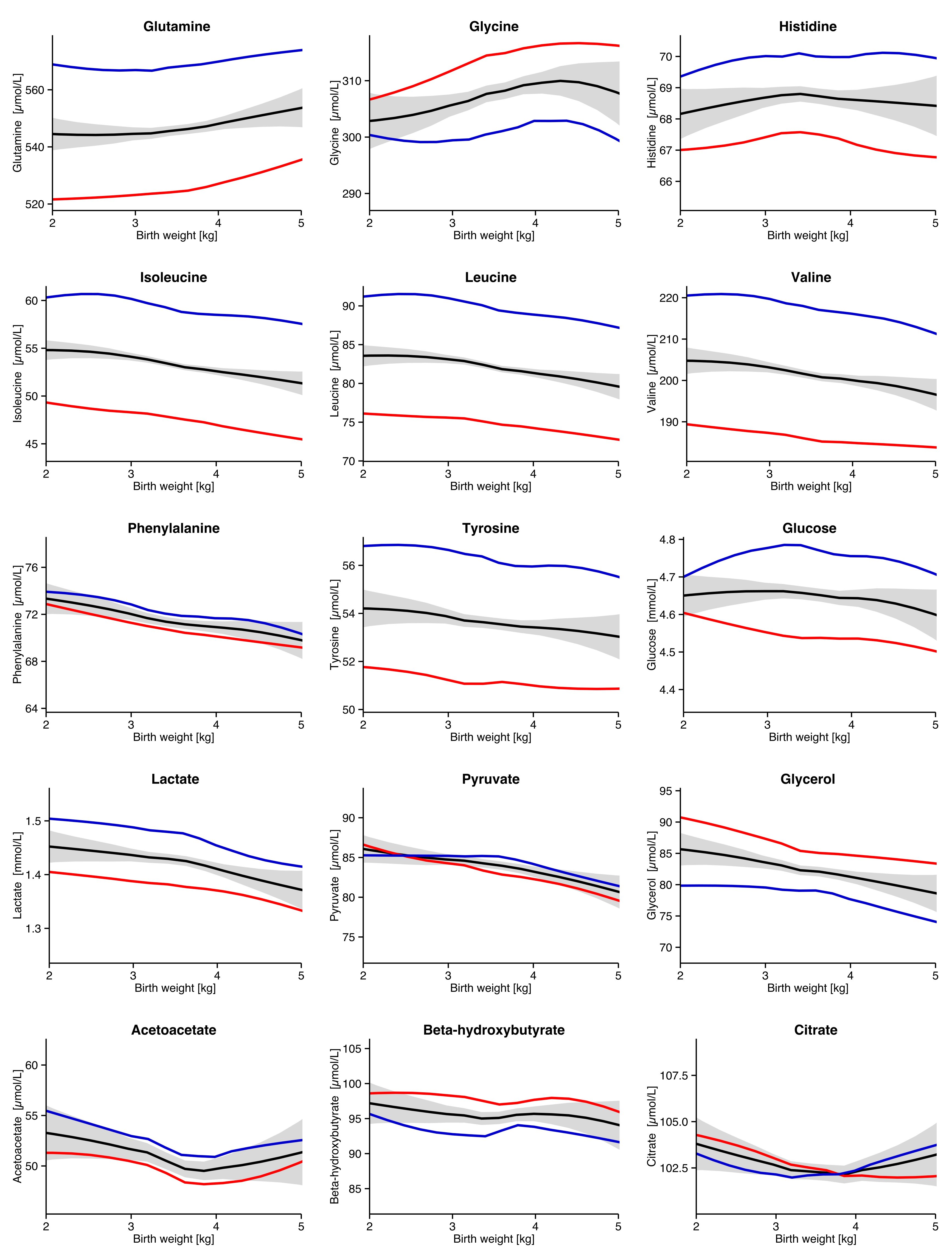


Continuous shape of the association between birth weight and 87 metabolic measures assessed in adulthood. Black curves denote the association shape for men and women combined (n=17 026), with the grey shaded area denoting the 95% confidence interval of the fit. Association shapes are depicted in blue for men (n=8336) and in red for women (n=8690). The association shapes were derived using local quadratic regression fitting evaluated at 25 points through the whole birth weight range. The metabolic measures were first adjusted for age and sex in each of the non-twin cohorts, and then pooled by combining the residuals and scaling back to absolute concentration units. Equivalent analyses were done stratified by sex. The 2 twin cohorts (n=1262) were omitted from these analyses due to their markedly lower distribution of birth weight (cf. Figure S1).

**Figure S9F. Curvilinear shapes of metabolic associations with birth weight.**


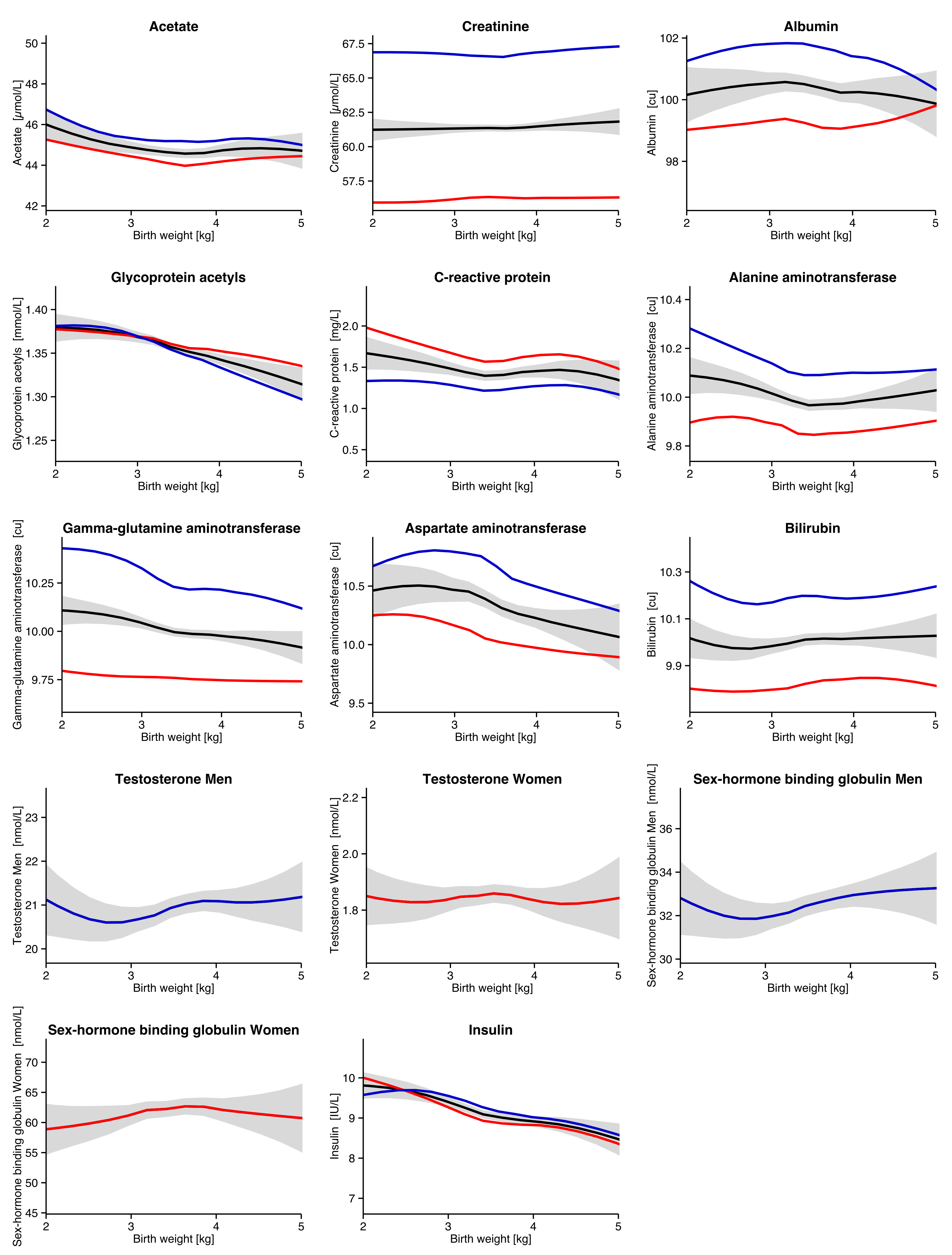


Continuous shape of the association between birth weight and 87 metabolic measures assessed in adulthood. Black curves denote the association shape for men and women combined (n=17 026), with the grey shaded area denoting the 95% confidence interval of the fit. Association shapes are depicted in blue for men (n=8336) and in red for women (n=8690). The association shapes were derived using local quadratic regression fitting evaluated at 25 points through the whole birth weight range. The metabolic measures were first adjusted for age and sex in each of the non-twin cohorts, and then pooled by combining the residuals and scaling back to absolute concentration units. Equivalent analyses were done stratified by sex. The 2 twin cohorts (n=1262) were omitted from these analyses due to their markedly lower distribution of birth weight (cf. Figure S1).
